# Phase resetting in human stem cell derived cardiomyocytes explains complex cardiac arrhythmias

**DOI:** 10.1371/journal.pcbi.1013935

**Published:** 2026-02-04

**Authors:** Khady Diagne, Thomas M. Bury, Morgan E. Pettebone, Marc W. Deyell, Zachary Laksman, Alvin Shrier, Leon Glass, Gil Bub, Emilia Entcheva

**Affiliations:** 1 Department of Quantitative Life Sciences, McGill University, Montreal, Canada; 2 Department of Physiology, McGill University, Montreal, Canada; 3 Department of Biomedical Engineering, George Washington University, Washington, District of Columbia, United States of America; 4 Division of Cardiology and Centre for Cardiovascular Innovation, University of British Columbia, Vancouver, Canada; UCI BME: University of California Irvine Department of Biomedical Engineering, UNITED STATES OF AMERICA

## Abstract

Phase resetting of cardiac oscillators underlies some complex arrhythmias. Here we use optogenetic stimulation to construct phase response curves (PRC) for spheroids of human induced pluripotent stem cell derived cardiomyocytes (hiPSC-CM) and a computational cardiomyocyte model to identify ionic mechanisms shaping the PRC. The clinical utility of the human PRCs is demonstrated by adding a patient-based conduction delay to the same equations to explain complex multi-day Holter ECG dynamics and cardiac arrhythmias. Periodic stimulation of these patient-based models and the computational model of human iPSC-CM reveal similar bifurcation patterns and entrainment zones. Cell therapy by injecting iPSC-CM into diseased hearts can induce ectopic foci-based engraftment arrhythmias. The PRC analysis offers a potential strategy to entrain these foci in a parameter space that avoids such arrhythmias.

## Author summary

Biological rhythms are responsible for key life functions such as the heartbeat, breathing, sleeping, and reproduction. In this work, we study the effects of stimuli on the heart’s rhythm. A single stimulus delivered to a spontaneously beating cluster of human cardiomyocytes, derived from stem cells, changes the timing of subsequent beats. Using experimental data, we have developed a mathematical model predicting the response of human heart cells to periodic stimuli. We then use these insights to analyze the mechanism of a type of cardiac arrhythmia (parasystole) that results from the interaction between the normal heart rhythm and a secondary abnormal pacemaker. By extending and personalizing the mathematical model, we can simulate the heart rhythms in human patients over extended periods of time. These results are also of interest in the context of a major complication that arises during an emerging therapy in which beating human cardiac cells are transplanted into damaged hearts. During early stages following the transplantation, there are often dangerous heart rhythms - engraftment arrhythmias. By combining our insights concerning resetting of cardiac oscillators with an ionic model of heart cells, we suggest possible strategies to control such arrhythmias.

## Introduction

Biological oscillations can be reset and entrained by appropriate stimuli [1–4]. A key strategy to understand and model the effects of stimuli on biological rhythms involves the measurement of the phase response curve (PRC) which quantifies the effects of a single stimulus on the oscillation as a function of its phase. Once the PRC is determined, we can compute the effects of periodic stimuli, assuming the oscillation is re-established rapidly following each stimulus. This insight was initially applied to study the effects of periodic stimuli to nerve cells [5]. Subsequently, these techniques have been applied in the analysis of diverse problems such as the effects of light on the circadian rhythm [6,7] and the effects of periodic stimuli on the cardiac rhythm [8,9].

PRCs are relevant to the analysis of parasystole – cardiac arrhythmia that arises when a cardiac region with automaticity competes with the native pacemaker (the sinus node) [8,10–13]. In parasystole, the abnormal focus is the source for premature ventricular complexes (PVCs), which are evident on the electrocardiogram [14]. However, since the PRC of human cardiac tissue has not been previously directly measured, theoretical work often assumes a PRC based on animal models [10–12] or that is inferred based on theoretical analysis [15–17].

We propose that analysis of parasystole will be also useful to optimize an emerging therapy for cardiac regeneration involving the transplantation of human induced pluripotent stem-cell derived cardiomyocytes (hiPSC-CM) into a diseased heart, an approach that has moved to clinical trials [18–20]. From a dynamics perspective, the transplantation of spontaneously beating hiPSC-CM into the heart can create an environment analogous to parasystole. A major complication associated with this therapy is the occurrence of potentially fatal cardiac rhythms, termed engraftment arrhythmias. These have been observed in large animal models within the first two weeks post-transplantation (injection) [19,21–23]. Electrocardiographic records from such studies show that at least in part, engraftment arrhythmias may result from competing oscillators (the sinus node and the transplantation site) [22,24,25].

In previous work, the dynamical responses of spontaneously beating cardiac tissue from embryonic chicks [9] and dogs [26] to external periodic stimulation have been represented by finite difference equations, describing phase resetting. Here, we sought to extend this PRC-based analysis to capture the dynamics of hiPSC-CM using experimental and computational approaches to better understand the potential role of phase resetting in the generation of PVCs in patients following stem cell grafts as well as in patients with frequent PVCs. We first experimentally obtain the PRC of human stem cell derived cardiac spheroids through optogenetic pacing [27]. We then adopt this empirically derived PRC to model PVC dynamics in clinical records. We further analyze the response of both the human iPSC-CM spheroids and the patient data to periodic stimulation. The developed iterative computational model capture faithfully the observed dynamics in the experimental and the clinical settings. To identify the molecular mechanisms shaping the PRCs and the dynamic responses, we construct the PRC for an ionic cell model of a hiPSC-CM [28] for a range of parameters. Finally, we dissect the phase-locking zones for the ionic, experimental and clinical models with the goal of developing strategies and interventions to reduce the PVC burden clinically.

## Results

### Phase response curves for hiPSC-CM spheroids

**Fig 1:**
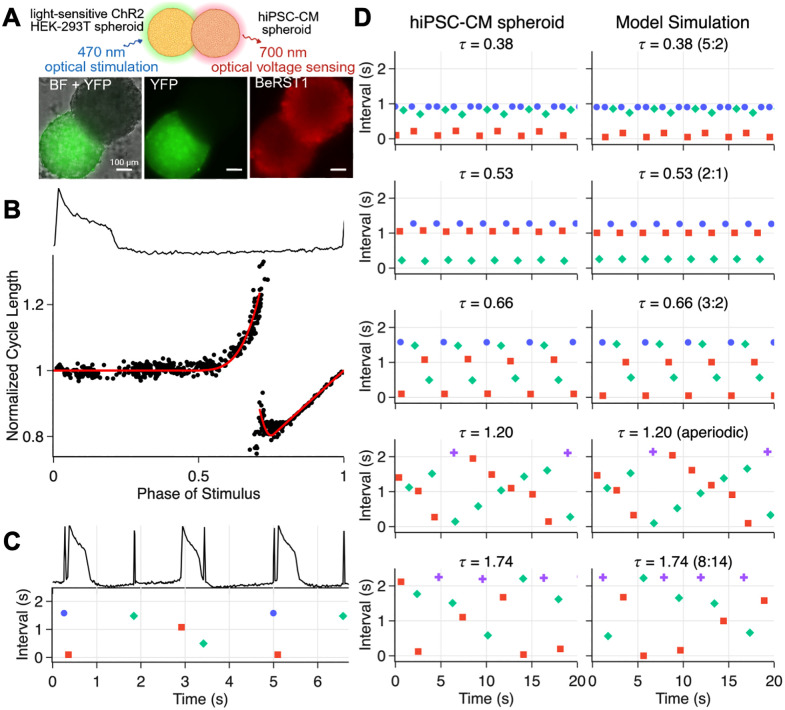
Phase response curve for human iPSC-CMs spheroids. **(A)** All-optical interrogation of cell spheroid pairs, shown in brightfield (BF), YFP fluorescence (YFP is a reporter tag for the optogenetic actuator ChR2), and BeRST1 (optical voltage sensor). **(B)** Trace of a full action potential cycle (top) and the phase-response curve (bottom), showing normalized cycle length as a function of the phase of the stimulus within the intrinsic cycle (2.4 s). Black dots show experimental data; the red line is the best fit of a piecewise-defined polynomial equation (S1 Text, RMSE = 0.056). **(C)** Dot plot showing intervals between stimuli of the HEK spheroid and APs of the hiPSC-CM spheroid. Colors and marker shapes denote interval type: stimulus-stimulus (blue circles); stimulus-AP (red squares); AP-stimulus (green diamonds); AP-AP (purple crosses). **(D)** Entrainment patterns observed for various pacing frequencies in the experiment (left) and the periodically-forced oscillator model (right). τ  is defined as the ratio of the stimulus cycle length to the intrinsic cycle length of the hiPSC-CMs. For periodic rhythms, locking ratios are given in parentheses in the form N:M, denoting N stimuli per M cycles of the forced oscillator.

To obtain a PRC of human-derived tissue, we used self-assembled spontaneously beating spheroids of hiPSC-CM paired with light-responsive spheroids of non-beating human embryonic kidney cells to form a tandem cell unit [27,29], Fig 1A. Delivery of a blue light stimulus leads to a depolarizing current in the light-responsive spheroid that propagates to the hiPSC-CM spheroid coupled to it. We stimulated the light-responsive spheroid with periodic depolarizing light pulses at well-defined frequencies which excited the hiPSC-CM spheroids. Using this method to stimulate the hiPSC-CM triggers a more physiological activation of the cells and is less damaging to the cells as compared to direct electrical stimulation; furthermore it does not require genetic modification of the cardiomyocytes [30]. Electrical responses (action potentials, APs) evoked in the whole hiPSC-CM spheroids were measured optically using a voltage-sensitive dye [31–33]. This all-optical electrophysiology approach is scalable and allows for long-term, contactless interrogation of the hiPSC-CM spheroids [32].

Let T  denote the spontaneous cycle length of the spheroid and $\tau =T_{\text{Stim}}/T$ be the normalized pacing cycle length, where $T_{\text{Stim}}$ is the period of external stimulation. The light stimuli were 10 ms and delivered at multiple values of τ . For each stimulus *i,* delivered at phase $\phi_{i}$, we measure the change in cycle length in the hiPSC-CM spheroid. Sweeping over multiple stimulation frequencies allows the full range of phases to be sampled, generating the PRC for the hiPSC-CM spheroid (Fig 1B). The resultant dynamics are shown in Fig 1C by plotting the intervals between consecutive events using colored dots. We fit a piecewise-defined polynomial equation g(ϕ) to this experimental PRC (Fig 1B and S1 Text). The spread of points around this red curve (RMSE = 0.056) reflects small long-term effects of the stimuli, measurement noise, and intrinsic biological noise. We use this equation to model the hiPSC-CM spheroid’s response to periodic stimulation [5,34]. Let $\phi_{i}$ be the phase of the *i*-th stimulus in the oscillator’s cycle. Then the phase of the next stimulus is given by


$$\phi_{i+\text{1}}=f\left(\phi_{i}\right)\;=\phi_{i}+\tau -g\left(\phi_{i}\right)\;(\text{mod}\;\text{1})$$ (1)

Simulation of this model corroborates the experimental observations, Fig 1D. The dynamics are periodic with period N $\phi_{i}=\phi_{i+N}$ and $\phi_{i+j}\neq \phi_{i}$ for 0<j<N; N:M periodic rhythms represent a repeating sequence of N stimuli and M action potentials. These experimental findings are consistent with the behavior of a perturbed oscillator (Eq. 1), with a strongly attracting limit cycle, i.e., following each stimulus, the cycle is reestablished, perhaps with a shift of phase.

Similar PRCs were obtained from six different stimulated hiPSC-CM spheroids (S1 Fig and S1 Table). The spontaneous frequency varied between 0.33 to 1.26Hz, with larger spheroids (spheroid pairs) beating slower. During the course of the experiment to obtain a full PRC (approximately 1 hour or longer per spheroid), the spontaneous frequency across the six pairs varied by about 18.5%. The discontinuity point in the constructed PRCs ranged between 0.38 and 0.71, S1 Table. Despite variability, these human cardiac PRCs have some common features: (i) A well-defined discontinuity separating cycle prolongation from shortening; (ii) Stimuli delivered early in the cycle have negligible effect on the cycle length or increase it; (iii) Stimuli delivered after the discontinuity lead to cycle shortening, with a “hook” followed by a distinctly linear segment. These features are consistent with previously reported experimental [9,26] and theoretical [35,36] studies of PRCs of non-human cardiac oscillators. The “hook” in the human cardiac PRCs obtained here, immediately past the discontinuity, was more pronounced than in previous studies from different species. Near the discontinuity (phase $\phi_{r}$), a stimulus either delays or triggers the next beat. This may reflect the influence of stochastic fluctuations due to ion channel behavior [35] or due to unrelated noise.

### Modelling dynamics of PVCs in clinical records using the experimentally derived PRC

Multi-day Holter recordings from 53 patients with more than 5% PVCs enrolled in the British Columbia PVC Registry were used. We developed a model of modulated parasystole incorporating the experimentally derived PRC and data-driven techniques to fit the model to individual patient ECG recordings (see S2 Text). The empirical PRC helped constrain the number of free parameters in the model in a biologically-grounded manner.

The clinical data was initially screened to select patients in whom more than 80% of the PVCs had the same morphology. This criterion yielded a subset of 40 patients. Identification of cycling coupling intervals — a hallmark of parasystolic dynamics [14]– further selected 7 out of the 40 patients (S2–S3 Figs). For these patients, we investigated the extent to which the modulated parasystole model could reproduce the clinically observed dynamics.

**Fig 2:**
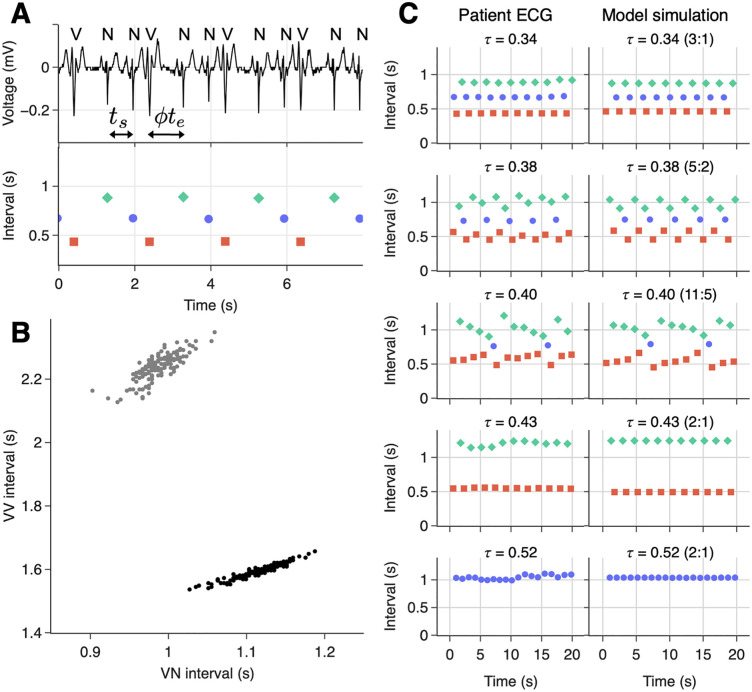
PVC dynamics in a patient with frequent PVCs and corresponding simulations of the modulated parasystole model. **(A)** A segment of the patient’s electrocardiogram (top) and associated inter-beat intervals (bottom). N denotes a sinus beat, V an ectopic beat (premature ventricular complex, PVC). The sinus cycle length is denoted by t_s_, the ectopic cycle length by t_e_ and *ϕ* is the phase of the sinus beat in the ectopic cycle. Intervals are color-coded: NN (blue circle), NV (red square), and VN (green diamond). **(B)** A representation of the phase response curve of the ectopic focus, inferred from sequences of bigeminy (alternating sinus and ectopic beats, black dots) and trigeminy (two sinus beats between ectopic beats, gray dots) (see S2 Text). **(C)** Sections of patient ECG recordings (left) and simulations of the modulated parasystole model (right), at five different ratios between sinus and ectopic cycle lengths ($\tau =t_{s}/t_{e}$). The locking ratio of the model is given in parentheses in the form N:M, denoting N sinus cycles per M ectopic cycles.


The modulated parasystole model builds on earlier work [12] by including a conduction delay into and out of the ectopic focus [37]. The model assumes a sinus pacemaker with period *t*_*s*_, an ectopic pacemaker with period *t*_*e*_, a refractory period *θ*, and modulation of the ectopic pacemaker by sinus beats via a PRC (*ϕ*). Sinus and ectopic beats are taken as instantaneous events that occur at fixed times. Sinus beats reset the ectopic pacemaker according to the PRC (*ϕ*). Ectopic beats are assumed to not reset the sinus pacemaker due to a refractory block at the AV node. As observed clinically, the model assumes that sinus beats immediately following an ectopic beat are blocked, and *vice versa*. Let *ϕ*_*i*_ be the phase of the *i*^th^ sinus beat in the ectopic cycle, determined by the timing of the sinus beat relative to the previous ectopic beat (Fig 2A). Then the subsequent phase is given by:

$$\phi_{i+\text{1}}=\;\left\{ \begin{array}{cc} \phi_{i}+\;\frac{t_{s}}{t_{e}}\;\;\;(\text{mod}\;\text{1}) & \text{0}\;\leq \phi_{i}<\frac{t_{s}-\;\theta }{t_{e}},\\ \phi_{i}+\;\frac{t_{s}}{t_{e}}+\text{1}-g\left(\phi_{i}+\;\frac{t_{lag}}{t_{e}}\right)\;\;(\text{mod}\;\text{1}) & \frac{t_{s}-\;\theta }{t_{e}}\leq \phi_{i}<\text{1},\; \end{array}\right.\;$$ (2)

where *t*_lag_ is the total conduction delay into and out of the ectopic focus, and other parameters are as previously defined. The shifted phase *ϕ* + *t*_lag_/*t*_*e*_ represents the phase at which the sinus beat stimulus arrives at the ectopic focus (S4 Fig). Accounting for this conduction delay is necessary to reproduce the dynamics observed in clinical recordings.

The model includes 10 parameters (S2 Table), which are fit to each patient (see S2 Text). We can reconstruct part of the hypothesized ectopic pacemaker’s PRC using the ECG (Figs 2B and S5) [17]. For the model, however, g(ϕ) is obtained from the experimentally derived PRC which spans the entire oscillator’s cycle. Three parameters are estimated directly from the clinical data: the sinus period *t*_*s*_, the refractory period *θ*, and the slope of the PRC after the discontinuity *S* (S6–S7 Figs). The remaining three parameters: ectopic cycle length *t*_*e*_, conduction delay *t*_lag_, and PRC discontinuity *ϕ*_*r*_, are chosen to minimize the mean absolute error between the model output and the clinical recordings. S3 Table shows the unique set of fitted parameters for each patient.

In Fig 2C we show dynamics from a single patient alongside model simulations generated using the best-fit parameters for five representative values of the sinus cycle length. We observe qualitatively different dynamics at each sinus cycle length, all of which are captured by the model. From the model, we can infer the locking ratio between the sinus and ectopic pacemakers. We observe simple ratios such as 2:1 and 3:1 locking, as well as higher-order ratios such as 11:5, which produce dynamics with a longer period. Notably, 2:1 locking occurs at both τ =0.43 and τ =0.52. At τ =0.52, , the ectopic beats fall within the refractory period and are therefore blocked, resulting in purely sinus rhythm. In contrast, at the shorter sinus cycle length corresponding to τ =0.43, the sinus beats land earlier in the ectopic cycle, allowing the ectopic beat to fall outside the refractory period and be expressed. This leads to alternating sinus and ectopic beats.

The model’s ability to reproduce dynamics over the entire recording was assessed using the Kolmogorov–Smirnov test [38], i.e., a good fit (*p* > 0.05) was considered when the inter-beat interval distribution from the simulation was not significantly different from that of the patient record. Allowing for a ± 5% variation in the fitted parameters, the proportion of the recording that was well reproduced by the model ranged from 63.4% to 95.0% across patients (S3 Table).

### Empirical bifurcation analysis

**Fig 3:**
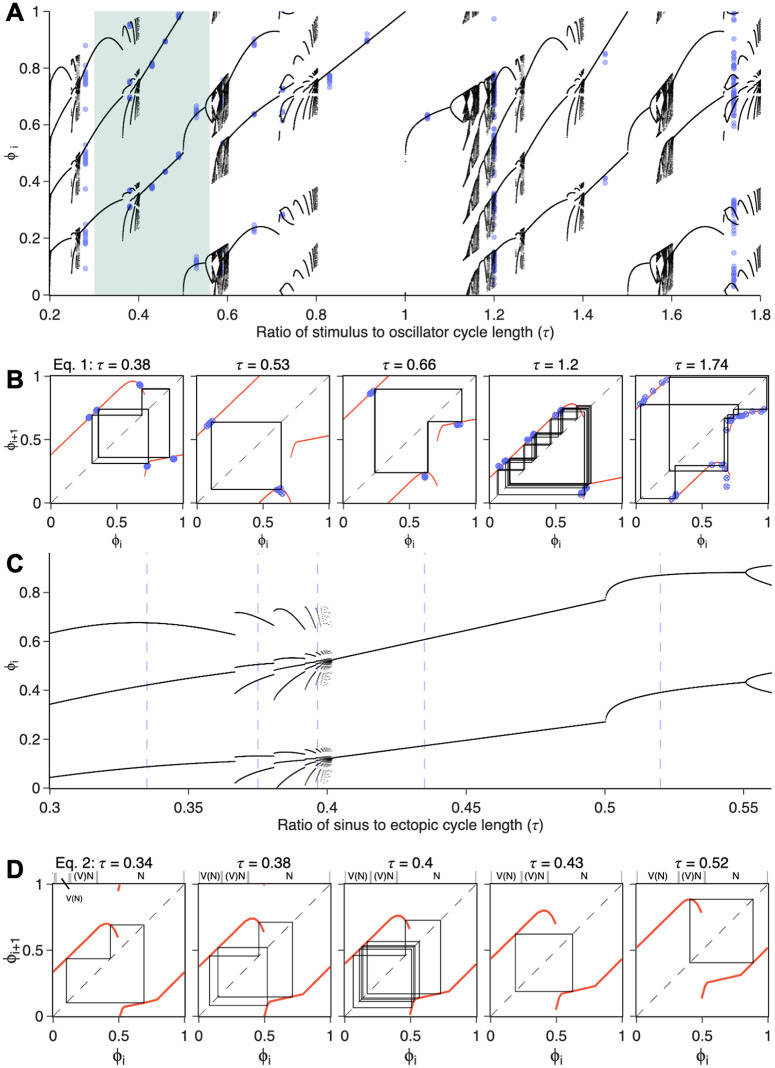
Dynamical regimes observed in experimental and clinical data, explained by bifurcation analysis of mathematical models. **(A)** Bifurcation diagram of the periodically-forced oscillator model (Eq. 1) using the experimentally fitted PRC from Fig 1B. Blue dots mark experimental data. The shaded region indicates the range of τ  values observed in the patient data. **(B)** Cobweb plots for Eq. 1 at selected values of τ  studied in experiments. The return map is shown in red; model iterations after transients are shown in black; experimental data are overlaid in blue. The identity line, along which $\phi_{i+\text{1}}=\phi_{i}$, is shown as a dashed line. **(C)** Bifurcation diagram of the modulated parasystole model (Eq. 2), using the same experimentally fitted PRC, plotted over the range of τ  values observed in the patient record. Vertical blue lines indicate specific τ  values corresponding to the clinical segments shown in Fig 2C. **(D)** Corresponding cobweb plots for Eq. 2 for each τ  value. The three regions above the graph indicate different contexts for the sinus beat depending on its phase. V(N), blocked sinus beat preceded by an ectopic beat; **(V)**N, sinus beat preceded by a blocked ectopic beat; N, single sinus beat. S8 Fig provides an explanation how the cobweb plots in panels B and D relate to the entrainment patterns seen in Figs 1 and 2.

We use the empirical PRC in Fig 1B to simulate the dynamics in a periodically stimulated hiPSC-CM spheroid (Eq. 1) and patient ECGs with recurrent PVCs (Eq. 2). We perform a bifurcation analysis of these models to explore the dynamical landscape of these systems (Fig 3). From iteration of Eq. 1, we compute the bifurcation diagram, i.e., the long-term stimulus phases as a function of τ for 0 ≤  τ ≤ 1.8 (Fig 3A). The diagram reveals a variety of stable periodic behaviors, interspersed with chaotic regions. A good agreement is seen with the experiments (overlayed in blue), despite noise. In Fig 3B, we plot the experimental results from Fig 1C in a return map of the phase of the stimulus in the oscillator’s cycle length in addition to the model’s iterations. This comparison allows us to distinguish between noisy periodic dynamics (τ = [0.38, 0.53, 0.66, 1.74]) and chaotic rhythms (τ = 1.2). The shaded area in the bifurcation diagram of Fig 3A highlights the range of 𝜏 values seen in the patient. We plot in Fig 3C the bifurcation diagram for the parasystole model in Eq. 2 for 0.3 ≤  𝜏 ≤ 0.55, which follows very similar bifurcations as those seen in the simpler model above. In this range, small variations in the sinus cycle length result in very different entrainment rhythms (Fig 3D), as seen in the clinical patient (Fig 2C). S8 Fig further clarifies the links between the return maps in Fig 3B and 3D and the entrainment patterns in Figs 1 and 2.

These simple reductionist methods highlight universal properties of periodically stimulated oscillators in experiments and people. The arrhythmias generated by an ectopic focus in the heart display a wide range of dynamics that only become obvious when considering the bifurcation analysis based on the PRC. The analysis not only describes the dynamics but also suggests ways to manipulate these rhythms to obtain simple locking regions in which ectopic firing is reduced or abolished. In the following sections, we explore biophysical methods to modify the PRC and how these modifications can be used to reduce ectopic firing across the models.

### Ion current contributors to the PRC in a computational model of hiPSC-CM

**Fig 4:**
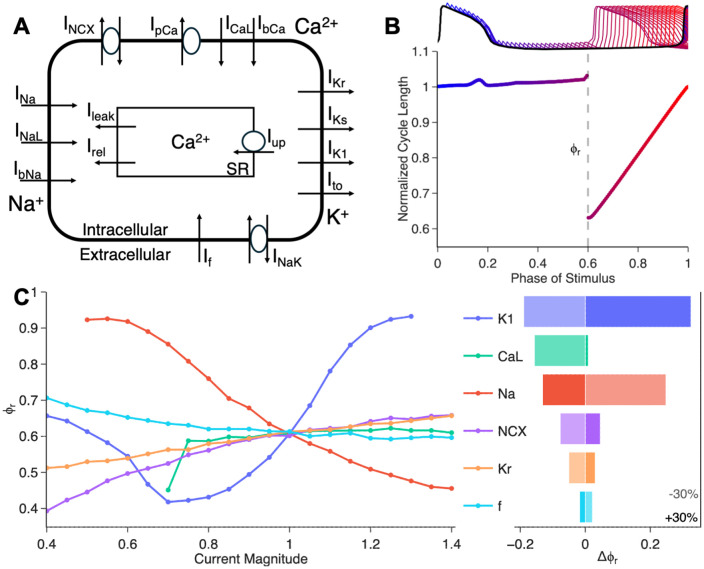
Phase resetting in a computational model for human iPSC-CMs. **(A)** Schematic representation of the ionic currents in the human iPSC-CM model (Paci et al, 2020). **(B)** Top: Trace of a full action potential cycle (black), overlaid with action potentials evoked by stimuli applied at incrementally increasing times (every 30 ms; traces color-coded by stimulus phase). Bottom: Corresponding phase response curve (PRC). **(C)** Sensitivity of the PRC discontinuity ($\phi_{r}$) to ionic current magnitudes (magnitude of 1 corresponds to default value). Left: $\phi_{r}$ as a function of current magnitude. Right: Change in $\phi_{r}$ following a ±30%  change in the conductance of each current (shaded: -30%, opaque: + 30%).

We carry out simulations using a computational model of a hiPSC-CM [28]. The ionic currents modelled are described in Fig 4A. To construct the PRC, in an AP cycle we deliver a stimulus of 10 ms and 1.5 × 10^−10^ A, probing the cycle at 10 ms increments (Fig 4B). While most stimuli delivered in the first 70% of the cycle have minimal effect, stimuli delivered early in the resting phase lead to a cycle length prolongation. At phases greater than about 0.7, there is an abrupt transition (discontinuity) in response to stimuli; these stimuli trigger an AP. This transition is associated with an all-or-none AP response as a critical voltage is crossed [35].

The location of the discontinuity $\phi_{r}$ plays a crucial role in determining the effects of periodic stimulation; decreasing $\phi_{r}$ increases the range of stimulus frequencies that lead to simple locking patterns. We examined the role of several ionic currents in the location of this discontinuity, Fig 4C. In the left panel, each current was varied in strength from 0.4x to 1.4x the default conductance magnitude in the model (1x). The fast sodium current I_Na_ has a strong effect on the PRC, decreasing $\phi_{r}$ as the conductance of I_Na_ is increased. The inward rectifier current I_K1_ has a powerful non-monotonic effect on the PRC. An increase from default values can prevent early triggering, while a decrease can significantly facilitate early triggering. Further decrease in I_K1_, however also prevents AP triggering. This is consistent with the complex and context-dependent role of I_K1_ on excitability [39]. Increases in the L-type calcium current I_CaL_, the Na/Ca exchanger current I_NCX_, the rapid delayed rectifier I_Kr_, and the funny/pacemaking current I_f_ have little effect on $\phi_{r}$. However, reducing any of these currents decreases $\phi_{r}$, except for I_f_ which has small effect in the opposite direction. In the right panel of Fig 4C, we show the change in $\phi_{r}$, at + or – 30% conductance for each current. Overall, the most significant currents in determining the location of the discontinuity in this model are I_K1_, I_Na_, and I_CaL_. Furthermore, the *in silico* PRC simulations corroborate that lower temperature, higher stimulus strength, and longer stimulus duration decrease $\phi_{r}$, helping trigger an early AP (S9 Fig). Note that for weak or short stimuli, there is no apparent discontinuity in the PRC.

### Entrainment zones infer strategies to suppress PVCs

**Fig 5:**
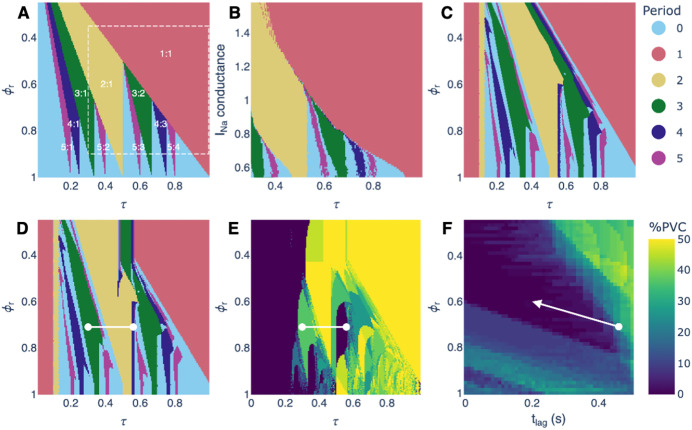
Locking zones across different models and an implied strategy for suppressing PVCs. (**A)** Locking zones in the oscillator model with a piecewise linear PRC, shown as a function of τ and $\phi_{r}$. Inset numbers indicate the locking ratio N:M, and colors represent the period N; “Period 0” refers to aperiodicity or N >5. The dashed box highlights the region also examined in the ionic model. **(B)** Locking zones in the ionic model as a function of sodium channel conductance. Similar locking regions appear in the oscillator model with a linear PRC (dashed box). **(C)** Locking zones in the oscillator model using the empirical PRC. **(D)** Locking zones in the modulated parasystole model, using the same PRC and other parameters derived from patient data. The white line indicates the range of 𝜏 values observed in the patient record. **(E)** Percentage of PVCs observed in the modulated parasystole model fit with patient data. **(F)** Percentage of PVCs averaged over 𝜏, shown as a function of $\phi_{r}$ and $t_{lag}$ (conduction time into and out of the ectopic focus). The white dot marks parameter values fit to the patient. The white arrow indicates the direction associated with a reduced PVC burden.

Fig 5 illustrates entrainment zones across the different models, and how they can guide strategies to suppress premature ventricular complexes (PVCs). For the periodically-forced oscillator model (Eq. 1) with a piecewise linear PRC (an approximation to the PRC of the hiPSC-CM computational model, see materials and methods), distinct entrainment zones arise across the τ -$\phi_{r}$ parameter space (panel A). Notably, smaller values of $\phi_{r}$ result in wider low-period locking zones. Similar entrainment zones are present in the hiPSC-CM computational model, where varying sodium channel conductance has a similar effect (panel B). When the oscillator model is extended to include the experimentally derived PRC (panel C) and then incorporated into a modulated parasystole model with patient-derived parameters (panel D), the global structure of the locking zones persists with slight changes based on the nonlinearity introduced in the PRC. The white line indicates the range of τ  values observed in the patient record. The PVC burden is computed from simulations of the modulated parasystole model over the same parameter range (Panel E). Under the current value of $t_{\text{lag}}$ fitted to the patient, variations in $\phi_{r}$ alone do not provide a reduction in PVC burden. However, when $t_{\text{lag}}$ is reduced (panel F), decreasing $\phi_{r}$ leads to fewer PVCs, as low-period locking becomes favored. In this regime, entrainment of the ectopic focus suppresses PVCs by timing them to fall consistently in the refractory period of the heart. Movement in parameter space toward these low-period zones (indicated by the white arrow) suggests a potential strategy for PVC suppression. This raises the possibility that modifying the PRC, for example through pharmacological interventions, could shift the system into more favorable locking zones and thereby reduce ectopic activity.

The locking zones in these models illustrate how small changes in parameter values can lead to dramatic changes in dynamics. To reproduce the observed rhythms across a patient record, it was necessary to allow parameters in the model to vary, consistent with the notion that physiological factors such as conduction velocity, autonomic tone, and excitability fluctuate over the day. This non-stationarity may help explain why patients with frequent PVCs sometimes present without PVCs on the day of their scheduled ablation.

## Discussion

We provide experimental characterization of PRCs for hiPSC-CM spheroids, using optogenetic stimulation and optical mapping. These PRCs share morphological similarities with curves from chick embryo heart cells [40] and canine Purkinje fibers [11], confirming universality of such responses. PRCs from an *in silico* model of these hiPSC-cardiomyocytes reveal the contribution of various ionic currents to phase resetting. There are several important applications of the empirically derived PRCs and the developed parasystole models.

First, combining the empirical human cardiac PRC (from hiPSC-CM spheroids) with a model for modulated parasystole reproduced many of the complex rhythms observed in patients with recurrent PVCs. Given that parasystole is highly correlated with future occurrence of ventricular fibrillation in cardiomyopathy patients [41], and can lead to sudden cardiac death following cardiac ablation [42], identification of patients with parasystole should be useful for risk stratification. Further, in fitting the model to the patient ECG, we obtain an estimate for the conduction delay between sinus beats and activation of the ectopic site. This measure may be useful in estimating the site of origin and severity of the PVCs (Purkinje vs. other) [43,44] and predicting the effect of changes in conduction velocity due to factors such as interstitial fibrosis and/or non-cardiomyocyte cell types.

A second potential area of application of the PRC analysis is the engraftment of hiPSC-CM for heart regeneration. Engrafted hiPSC-CMs behavior is consistent with a stimulated oscillator [45]. At least eight clinical trials are underway in Japan, Germany and China to deliver hiPSC-CMs or engineered constructs of such cells in patients with heart failure or other cardiac pathology [18,19]. Engraftment of these cells in large animal models often was associated with transient engraftment arrhythmias within the first two weeks while the graft is loosely coupled to the donor heart. Large-scale tissue simulations focused exclusively on the graft-host electrical coupling affecting the engraftment arrhythmia propensity [24,25,46]. As such coupling strengthens over time, the engraftment arrhythmias subside. In addition, suppression of the ectopic activity from the graft reduced the incidence of lethal arrhythmias originating from this ectopic focus [22,47].

There is a desire to increase the safety of hiPSC-CM transplantation by engineering the graft to have anti-arrhythmic properties upon transplantation or by administering pharmacological therapy within the vulnerable window. Our PRC analysis and parasystole modeling offer insights on ion channel targets and can inform ongoing pharmacological treatments [47] and gene-editing approaches [22] to prevent engraftment arrhythmias. This can be done by revealing critical molecular targets to enlarge the simple locking entrainment zones (Figs 4, 5), by in depth exploration of the dynamics of the system (Fig 3), and by data-driven personalization for a particular patient (Fig 2) to devise rhythm control strategies. While large-scale 2D and 3D cardiac tissue modeling with patient-specific geometries is making strides in devising “digital twins” in clinical cardiology [48], longer-term dynamics simulations of rhythm disturbances require a different, computationally efficient approach, as showcased here, to fully leverage multi-day patient records for personalized diagnosis and treatment.

## Materials and methods

### Ethics statement

*In vitro* experiments with human iPSC-CMs were conducted at George Washington University under an approved Institutional Biosafety Committee (IBC) protocol IBC-21–131. For the ECG records, the use of data arising out of the CardioSTAT Holter monitors, and the protection of personal information with regard to the CardioSTAT that is used by each individual wearer of the CardioSTAT was approved by the University of British Columbia Research Ethics Board and is in compliance with its policies. For this study, anonymized data was used and therefore no consent was obtained.

### Cell spheroid assembly and pairing

To collect experimental PRCs from human iPSC-CMs we used a two-spheroid model, where an optogenetically-responsive cell spheroid was coupled to a spheroid of unmodified human iPSC-CMs and light pulses were used to perturb the system, while the generated responses (action potentials) were measured optically.

We previously developed an immortalized 293T-HEK cell line expressing ChR2 tagged with yellow fluorescent protein (YFP) [27] using the Addgene construct pcDNA3.1/hChR2(H134R)-eYFP (from Karl Deisseroth). ChR2-HEK cells were cultured in Dulbecco’s Modified Eagle Medium (DMEM, ATCC) with 10% fetal bovine serum (FBS), 1% 200mM L-Glutamine, and 1% penicillin-streptomycin. After trypsinization, using 0.05% trypsin-disodium ethylenediaminetetraacetic acid (EDTA), spheroids were formed by plating the cell suspension at ~2.5 to 10 × 10^3^ cells per well in ultra-low-attachment, round bottom Corning 96-well (#CLS4920, Millipore Sigma) or 384-well (#CLS3830, Millipore Sigma) plates. Spheroids were typically used the following day.

Human induced pluripotent stem-cell-derived cardiomyocytes (hiPSC-CMs) were thawed following the manufacturer’s protocol (iCell^2^, #R1017, Fujifilm-Cellular Dynamics, with purified ventricular phenotype). The cells were suspended in plating media and seeded at about 5–10 × 10^3^ cells/well in ultra-low attachment, round bottom plates. 50% of iCell maintenance medium was replaced every 48 hours to maintain spheroid integrity.

One ChR2-HEK spheroid and one hiPSC-CM spheroid were carefully transferred to the same well in a separate Corning 384-well spheroid microplate, avoiding shear forces, and the paired spheroids were incubated for 3–24 hours to form electrical contact.

### Functional experiments with all-optical electrophysiology

Measurements were performed on an inverted Nikon Eclipse Ti2 microscope at 20 × magnification. The hiPSC-CM spheroids were labeled with the small-molecule, voltage-sensitive BeRST1 dye at a 2 µM concentration (generously provided by Evan W. Miller [33], following procedures established in previous studies [31]. Optogenetic stimulation was done through a digital micromirror device (DMD) (Polygon 4000, Mightex, Toronto, ON, Canada) using 470 nm blue light pulses at 10 ms duration, < 0.15 mW/mm^2^, at various frequencies to drive the hiPSC-CM spheroids while the illuminated region was covering the ChR2-HEK spheroid. For entrainment experiments, the same setup was used, but pulse durations were adjusted to 5, 10, or 20 ms. Optical voltage imaging with BeRST1 dye involved 660 nm excitation, with emitted light captured with a long pass filter at 700 nm using an iXon Ultra 897 EMCCD camera (Andor Technology Ltd., Belfast, United Kingdom), at 206 fps.

### Experimental protocols

To collect data for construction of the PRC, for each spheroid pair, a 40 sec recording of spontaneous activity was taken initially to calculate the hiPSC-CM spheroid’s spontaneous rate. Using this spontaneous rate, the stimulus frequency was defined by solving


$$R=\;\frac{\text{Spontaneous}\;\text{frequency}\;(\text{Hz})}{\text{Stimulus}\;\text{Frequency}\;(\text{Hz})}$$

where R is a user-defined ratio. A subsequent recording was then taken consisting of three phases: the first 40 sec captured spontaneous activity (no excitation), the next 10 min applied optical excitation to the stimulation region at the calculated frequency, and the final 40 sec recorded spontaneous activity to give the excitable cells time to recover between each tested frequency. Voltage activity was recorded from the sensing region of the hiPSC-CM spheroid for the entire duration. This process, including recalculating the spontaneous rate, was conducted for each value of R. After all tests, one final spontaneous recording was taken. These recordings were performed for n = 6 spheroid pairs with all optical pulses set to 10 ms durations.

To collect entrainment data, a similar protocol was used with some differences. Based on an initial 40 sec record of spontaneous frequency, 13–17 various pacing frequencies were applied - below, above, or close to the spontaneous rate. Each recording consisted of three phases: 30 sec of spontaneous activity, 1 min of optical excitation, then a final 30 sec of spontaneous activity for recovery between frequencies. Additional 40 sec spontaneous recordings were performed periodically to check the spontaneous rate and a final spontaneous recording was taken after all measurements were completed. These recordings were conducted on n = 1 spheroid pair with data collected at optical pulse durations of 5, 10, and 20 ms. Phase resetting data was also obtained for this spheroid pair at all three pulse durations using the methods described previously, with 5 min of optical stimulation recorded instead of 10min.

### Entrainment in an ionic model of a human iPSC-CM

We investigated the phase resetting curves (PRCs) from an ionic computational model of human cardiomyocyte by Paci et al., that was constrained by multiple experimental voltage and calcium records obtained by all-optical electrophysiology from the same ventricular-like hiPSC-CMs as used here [28]. This model follows the classic Hodgkin-Huxley formulation and includes the cytosol and sarcoplasmic reticulum compartments. The MATLAB implementation is made available by the authors. We simulate the model at 37˚C with external ionic concentrations of [Na^+^]_o_ = 151 mM, [K^+^]_o_ = 5.4 mM, and [Ca^2+^]_o_ = 1.8mM, and internal initial ionic concentrations of [Na^+^]_i_ = 9.2 mM, [K^+^]_i_ = 150 mM, [Ca^2+^]_i_ = 0.0002 mM, and [Ca^2+^]_SR_ = 0.32 mM. Keeping the default parameters of the model, we obtain a cycle length of 1.71 sec for the simulated cell.

For the locking zones in Fig 5A of the manuscript, we use a piecewise linear equation to model the PRC.


$$\text{g}(\phi )\;=\;\left\{ \begin{array}{cc} \text{1}\; & \text{0}\;\leq \;\phi <\phi_{r}\\ \phi & \phi_{r}\leq \phi <\text{1} \end{array}\right.\;$$

where $\phi_{r}$ is the phase at the discontinuity. This serves as a simple approximation to the PRC obtained in the ionic model. To compute the locking zones of the ionic model in Fig 5B, we simulate the Paci model using the Python package myokit [49] which employs the solver CVODES [50] for fast numerical integration of stiff ordinary differential equations.

## Supporting information

S1 Text Fitting a polynomial equation to experimental PRCs. (PDF)

S2 Text Parasystole and resetting in clinical ECGs. (PDF)

S1 Fig Phase-resetting curves for individual hiPSC-CM spheroids. The red line shows the fitted PRC function given by Eq. (1). Optimal parameters were obtained using a nonlinear least-squares algorithm (Trust Region Reflective method). Best-fit parameter values for each aggregate are listed in S1 Table. The inset shows the root mean squared error (RMSE) of each fit.
(PDF)

S2 Fig Filtering of clinical data based on the presence of a dominant PVC morphology and a cycling coupling interval. (PDF)

S3 Fig Sections of patient data with cycling coupling intervals (left) and corresponding model simulation (right). Each panel shows inter-beat intervals from six different patient records identified as exhibiting a cycling coupling interval. Intervals are color-coded as follows: blue for sinus–sinus, red for sinus–ectopic, green for ectopic–sinus, and purple for ectopic–ectopic beats. These sections are used to fit the model parameters *te*, *t*lag, and *ϕ*r for each record by minimizing the mean absolute error between the model simulation and the data.
(PDF)

S4 Fig Illustration of conduction time into and out of the ectopic focus. This ECG shows a bigeminal rhythm (alternating sinus beats and ectopic beats). Assuming the ectopic beats originate from an ectopic pacemaker, we define the phase *ϕ* as the time between the ectopic beat and the subsequent sinus beat divided by the natural period of the ectopic pacemaker *te*. We assume that there is a conduction time *t*out from the firing of the ectopic pacemaker to the time of the PVC.We also assume there is a conduction time *t* in from the time of the sinus beat to the modulation of the ectopic focus. The shifted phase *ϕ* + *t*lag/*t*e therefore represents the phase at which sinus stimulus arrives at the ectopic focus.
(PDF)

S5 Fig Notation for beat-to-beat intervals. (A) Trace shows a section of ECG from record AC5137 during bigeminy (one sinus beat in between ectopic beats). VN1 denotes the interval from the ectopic beat to the (first) sinus beat. VV0 denotes the intrinsic ectopic cycle length. VV1 denotes modified ectopic cycle length due to the sinus beat. (B) Trace shows a section of the same record in trigeminy (two sinus beats in between ectopic beats). VN1 denotes the interval from the ectopic beat to the first sinus beat. VN2 denotes the interval from the ectopic beat to the second sinus beat. VV0 denotes the intrinsic ectopic cycle length. VV1 denotes modified ectopic cycle length due to the first sinus beat. VV2 denotes the modified cycle length due to the first and second sinus beats combined.
(PDF)

S6 Fig Estimating the PRC resetting slope (S) from the ECG. (A) Inter-beat intervals for a 30 s section of ECG from record AC5137. Intervals are either between two sinus beats (blue), between a sinus and an ectopic beat (red) or between an ectopic and sinus beat (green). (B) The interval between two consecutive ectopic beats (VV) as a function of the interval between the first ectopic and intervening sinus beat (VN) during periods of NIB = 1 (a single sinus beat between two ectopic beats). The slope of the linear regression through the points provides an estimation for the slope of the resetting curve (S).
(PDF)

S7 Fig Estimating the refractory period (*θ*) from the ECG. (A) Distributions of the coupling interval (NV) at different values of the sinus cycle length (bins of width 0.1 s) for record AC5137. (B) Linear regression through the points that mark the 5th percentile of each distribution. The refractory period at a given sinus cycle length is taken as the coupling interval on this linear regression minus a fixed amount ϵ = 0.05 s. Let *m* and *c* be the slope and intercept of the linear regression, then (*t*_*s*_) = (*t*_*s*_ − *c*)/*m* −  ϵ.
(PDF)

S8 Fig Notation in Poincare´ Maps. (A) Poincare´ map from Fig 3B for 𝜏  = 0.66, showing a stable cycle of period 3. At this phase-locking pattern, the stimulus phase in the oscillator’s cycle alternates between *ϕ*_1_, *ϕ*_2_, and *ϕ*_3_. (B) Signal trace for the same experiment, showing the three phases of the stimulus in the phase-locked pattern. (C) Corresponding interbeat intervals, with the same notation as in Figs 1D and 2C.
(PDF)

S9 Fig Effects of temperature, stimulus strength, and duration on the PRC from the Paci et al. 2020 ionic model (A) Stimulus strength =  0.15 nA, stimulus duration = 10 ms, temperature from 21°C in indigo to 41°C in yellow. (B) stimulus strength from 0.05 nA in indigo to 0.19 nA in green, stimulus duration = 10 ms, temperature = 37^°^C. (C) stimulus strength = 0.15 nA, stimulus duration from 5 ms in indigo to 20 ms in blue, temperature = 37^°^C.
(PDF)

S1 Table Optimal parameters for fitting the PRC function (Eq. (1)) to the experimental PRCs shown in S1 Fig. Aggregate labels correspond to the panel labels in S1 Fig. The root mean squared error (RMSE) is used to assess the goodness of fit. The intrinsic cycle length (iCL) represents the range of spontaneous beat intervals.
(PDF)

S2 Table Parameters, definitions, and estimation methods for the modulated parasystole model. Ranges/values are across the 7 patients identified as having cycling coupling intervals given in S3 Table. PRC: phase response curve; s: seconds; –: dimensionless.
(PDF)

S3 Table Model parameter values determined for each patient, along with the proportion of 30-second segments of the ECG record that are well described by the model within a ±5% and ±10% variation in parameter values. The sinus cycle length (*t*_*s*_) is measured for each 30-second section of the record and given as a range. The refractory period (*θ*) is determined from a linear function of *t*_*s*_, derived from the distribution of coupling intervals across heart rates (S7 Fig). The ectopic cycle length (*t*_*e*_), the lag time (*t*_lag_), and the PRC discontinuity (*ϕ*_𝑟_) are determined by fitting the model to a 30-second section identified as having cycling coupling intervals. The slope of the PRC (*S*) is determined by the slope in the plot of VV intervals against VN intervals (S6 Fig). The model is considered a good fit if the Kolmogorov–Smirnov test does not detect a statistically significant difference (*p* > 0.05) between the interbeat interval distributions of the patient and the model.
(PDF)

## References

[1] Winfree AT. The geometry of biological time. New York, NY: Springer; 1980.

[2] Glass L, Mackey MC. From clocks to chaos: The rhythms of life. Princeton, NJ: Princeton University Press; 1988.

[3] Schultheiss NW, Prinz AA, Butera RJ. Phase response curves in neuroscience: theory, experiment, and analysis. New York, NY: Springer Science & Business Media; 2011.

[4] Strogatz SH. Sync: How order emerges from chaos in the universe, nature, and daily life. New York, NY: Grand Central Publishing; 2012.

[5] Perkel DH, Schulman JH, Bullock TH, Moore GP, Segundo JP. Pacemaker neurons: effects of regularly spaced synaptic input. Science. 1964;145(3627):61–3. doi: 10.1126/science.145.3627.61 14162696

[6] Duffy JF, Czeisler CA. Effect of light on human circadian physiology. Sleep Med Clin. 2009;4(2):165–77. doi: 10.1016/j.jsmc.2009.01.004 20161220 PMC2717723

[7] Bagheri N, Stelling J, Doyle FJ 3rd. Circadian phase resetting via single and multiple control targets. PLoS Comput Biol. 2008;4(7):e1000104. doi: 10.1371/journal.pcbi.1000104 18795146 PMC2536509

[8] Moe GK, Jalife J, Mueller WJ, Moe B. A mathematical model of parasystole and its application to clinical arrhythmias. Circulation. 1977;56(6):968–79. doi: 10.1161/01.cir.56.6.968 923066

[9] Guevara MR, Glass L, Shrier A. Phase locking, period-doubling bifurcations, and irregular dynamics in periodically stimulated cardiac cells. Science. 1981;214(4527):1350–3. doi: 10.1126/science.7313693 7313693

[10] Jalife J, Antzelevitch C, Moe GK. The case for modulated parasystole. Pacing Clin Electrophysiol. 1982;5(6):911–26. doi: 10.1111/j.1540-8159.1982.tb00030.x 6184694

[11] Jalife J, Moe GK. A biologic model of parasystole. Am J Cardiol. 1979;43(4):761–72. doi: 10.1016/0002-9149(79)90076-6 425913

[12] Courtemanche M, Glass L, Rosengarten MD, Goldberger AL. Beyond pure parasystole: promises and problems in modeling complex arrhythmias. Am J Physiol. 1989;257(2 Pt 2):H693-706. doi: 10.1152/ajpheart.1989.257.2.H693 2764150

[13] Diagne K, Bury TM, Deyell MW, Laksman Z, Shrier A, Bub G, et al. Rhythms from two competing periodic sources embedded in an excitable medium. Phys Rev Lett. 2023;130(2):028401. doi: 10.1103/PhysRevLett.130.028401 36706395

[14] Marcus GM. Evaluation and management of premature ventricular complexes. Circulation. 2020;141(17):1404–18. doi: 10.1161/CIRCULATIONAHA.119.042434 32339046

[15] Schulte-Frohlinde V, Ashkenazy Y, Goldberger AL, Ivanov PC, Costa M, Morley-Davies A, et al. Complex patterns of abnormal heartbeats. Phys Rev E Stat Nonlin Soft Matter Phys. 2002;66(3 Pt 1):031901. doi: 10.1103/PhysRevE.66.031901 12366146

[16] Schulte-Frohlinde V, Ashkenazy Y, Ivanov PC, Glass L, Goldberger AL, Stanley HE. Noise effects on the complex patterns of abnormal heartbeats. Phys Rev Lett. 2001;87(6):068104. doi: 10.1103/PhysRevLett.87.068104 11497867

[17] Takayanagi K, Nakahara S, Hori Y, Sakai Y, Taguchi I, Ikeda N. Ectopic cycle length estimation from the quantified distribution patterns of ventricular bigeminy and trigeminy. Heart Rhythm O2. 2021;2(2):138–48. doi: 10.1016/j.hroo.2021.03.003 34113916 PMC8183894

[18] Sugiura T, Shahannaz DC, Ferrell BE. Current status of cardiac regenerative therapy using induced pluripotent stem cells. Int J Mol Sci. 2024;25(11):5772. doi: 10.3390/ijms25115772 38891960 PMC11171475

[19] Yan W, Xia Y, Zhao H, Xu X, Ma X, Tao L. Stem cell-based therapy in cardiac repair after myocardial infarction: Promise, challenges, and future directions. J Mol Cell Cardiol. 2024;188:1–14. doi: 10.1016/j.yjmcc.2023.12.009 38246086

[20] Jebran A-F, Seidler T, Tiburcy M, Daskalaki M, Kutschka I, Fujita B, et al. Engineered heart muscle allografts for heart repair in primates and humans. Nature. 2025;639(8054):503–11. doi: 10.1038/s41586-024-08463-0 39880949 PMC11903342

[21] Shiba Y, Gomibuchi T, Seto T, Wada Y, Ichimura H, Tanaka Y, et al. Allogeneic transplantation of iPS cell-derived cardiomyocytes regenerates primate hearts. Nature. 2016;538(7625):388–91. doi: 10.1038/nature19815 27723741

[22] Marchiano S, Nakamura K, Reinecke H, Neidig L, Lai M, Kadota S, et al. Gene editing to prevent ventricular arrhythmias associated with cardiomyocyte cell therapy. Cell Stem Cell. 2023;30(5):741. doi: 10.1016/j.stem.2023.04.010 37146587 PMC10286102

[23] Romagnuolo R, Masoudpour H, Porta-Sánchez A, Qiang B, Barry J, Laskary A, et al. Human embryonic stem cell-derived cardiomyocytes regenerate the infarcted pig heart but induce ventricular tachyarrhythmias. Stem Cell Reports. 2019;12(5):967–81. doi: 10.1016/j.stemcr.2019.04.005 31056479 PMC6524945

[24] Gibbs CE, Marchianó S, Zhang K, Yang X, Murry CE, Boyle PM. Graft-host coupling changes can lead to engraftment arrhythmia: a computational study. J Physiol. 2023;601(13):2733–49. doi: 10.1113/JP284244 37014103 PMC10901678

[25] Gibbs CE, Boyle PM. Population-based computational simulations elucidate mechanisms of focal arrhythmia following stem cell injection. J Mol Cell Cardiol. 2025;204:5–16. doi: 10.1016/j.yjmcc.2025.04.010 40280466 PMC12162212

[26] Antzelevitch C, Bernstein MJ, Feldman HN, Moe GK. Parasystole, reentry, and tachycardia: a canine preparation of cardiac arrhythmias occurring across inexcitable segments of tissue. Circulation. 1983;68(5):1101–15. doi: 10.1161/01.cir.68.5.1101 6193902

[27] Jia Z, Valiunas V, Lu Z, Bien H, Liu H, Wang H-Z, et al. Stimulating cardiac muscle by light: cardiac optogenetics by cell delivery. Circ Arrhythm Electrophysiol. 2011;4(5):753–60. doi: 10.1161/CIRCEP.111.964247 21828312 PMC3209525

[28] Paci M, Passini E, Klimas A, Severi S, Hyttinen J, Rodriguez B, et al. All-Optical Electrophysiology Refines Populations of In Silico Human iPSC-CMs for Drug Evaluation. Biophys J. 2020;118(10):2596–611. doi: 10.1016/j.bpj.2020.03.018 32298635 PMC7231889

[29] Chua CJ, Han JL, Li W, Liu W, Entcheva E. Integration of engineered “spark-cell” spheroids for optical pacing of cardiac tissue. Front Bioeng Biotechnol. 2021;9:658594. 10.3389/fbioe.2021.658594 34222210 PMC8249938

[30] Entcheva E. Cardiac optogenetics. Am J Physiol Heart Circ Physiol. 2013;304(9):H1179-91. doi: 10.1152/ajpheart.00432.2012 23457014 PMC3652095

[31] Klimas A, Ortiz G, Boggess SC, Miller EW, Entcheva E. Multimodal on-axis platform for all-optical electrophysiology with near-infrared probes in human stem-cell-derived cardiomyocytes. Prog Biophys Mol Biol. 2020;154:62–70. doi: 10.1016/j.pbiomolbio.2019.02.004 30850184 PMC6728218

[32] Entcheva E, Kay MW. Cardiac optogenetics: a decade of enlightenment. Nat Rev Cardiol. 2021;18(5):349–67. doi: 10.1038/s41569-020-00478-0 33340010 PMC8127952

[33] Huang Y-L, Walker AS, Miller EW. A Photostable silicon rhodamine platform for optical voltage sensing. J Am Chem Soc. 2015;137(33):10767–76. doi: 10.1021/jacs.5b06644 26237573 PMC4666802

[34] Glass L, Guevara MR, Belair J, Shrier A. Global bifurcations of a periodically forced biological oscillator. Phys Rev A. 1984;29(3):1348–57. 10.1103/PhysRevA.29.1348

[35] Krogh-Madsen T, Glass L, Doedel EJ, Guevara MR. Apparent discontinuities in the phase-resetting response of cardiac pacemakers. J Theor Biol. 2004;230(4):499–519. doi: 10.1016/j.jtbi.2004.03.027 15363672

[36] Kowtha VC, Kunysz A, Clay JR, Glass L, Shrier A. Ionic mechanisms and nonlinear dynamics of embryonic chick heart cell aggregates. Prog Biophys Mol Biol. 1994;61(3):255–81. doi: 10.1016/0079-6107(94)90002-7 8073123

[37] Bury TM, Diagne K, Olshan D, Glass L, Shrier A, Lerman BB, et al. The inverse problem for cardiac arrhythmias. Chaos. 2023;33(12):123130. doi: 10.1063/5.0161210 38149994

[38] Virtanen P, Gommers R, Oliphant TE, Haberland M, Reddy T, Cournapeau D, et al. SciPy 1.0: fundamental algorithms for scientific computing in Python. Nat Methods. 2020;17(3):261–72. doi: 10.1038/s41592-019-0686-2 32015543 PMC7056644

[39] Langen JS, Boyle PM, Malan D, Sasse P. Optogenetic quantification of cardiac excitability and electrical coupling in intact hearts to explain cardiac arrhythmia initiation. Sci Adv. 2025;11(9):eadt4103. doi: 10.1126/sciadv.adt4103 40020054 PMC11870084

[40] Clay JR, Brochu RM, Shrier A. Phase resetting of embryonic chick atrial heart cell aggregates. Experiment and theory. Biophys J. 1990;58(3):609–21. doi: 10.1016/S0006-3495(90)82404-8 2207253 PMC1281002

[41] Do Duc H, O’Meara K, Lee J, Meyer S, Hanna P, Mori S, et al. Ventricular Parasystole in Cardiomyopathy Patients. JACC: Clin Electrophysiol. 2023;9(7_Part_1):936–48. doi: 10.1016/j.jacep.2022.11.014 37438043

[42] Koyanagi Y, Nakamura Y, Ishikawa T, Arai S, Asano T, Shinke T. Sudden death from long-standing benign ventricular parasystole after atrial fibrillation ablation. HeartRhythm Case Rep. 2025;11(8):824–8. doi: 10.1016/j.hrcr.2025.06.004 40896676 PMC12399124

[43] Haïssaguerre M, Duchateau J, Dubois R, Hocini M, Cheniti G, Sacher F, et al. Idiopathic Ventricular Fibrillation: Role of Purkinje System and Microstructural Myocardial Abnormalities. JACC: Clin Electrophysiol. 2020;6(6):591–608. doi: 10.1016/j.jacep.2020.03.010 32553208 PMC7308805

[44] Escande W, Gourraud J-B, Haissaguerre M, Gandjbakhch E, Lavergne T, Martins R, et al. Malignant Purkinje ectopy induced by sodium channel blockers. Heart Rhythm. 2022;19(10):1595–603. doi: 10.1016/j.hrthm.2022.06.034 35835363

[45] Huethorst E, Bishop MJ, Burton FL, Denning C, Gadegaard N, Myles RC, et al. Evidence for intermittent coupling of intramyocardial small, engineered heart tissues acutely implanted into rabbit myocardium. Cardiovasc Res. 2025;121(11):1697–711. doi: 10.1093/cvr/cvaf034 40036828 PMC12477673

[46] Yu JK, Liang JA, Weinberg SH, Trayanova NA. Computational modeling of aberrant electrical activity following remuscularization with intramyocardially injected pluripotent stem cell-derived cardiomyocytes. J Mol Cell Cardiol. 2022;162:97–109. doi: 10.1016/j.yjmcc.2021.08.011 34487753 PMC8766907

[47] Nakamura K, Neidig LE, Yang X, Weber GJ, El-Nachef D, Tsuchida H, et al. Pharmacologic therapy for engraftment arrhythmia induced by transplantation of human cardiomyocytes. Stem Cell Reports. 2021;16(10):2473–87. doi: 10.1016/j.stemcr.2021.08.005 34506727 PMC8514851

[48] Laubenbacher R, Mehrad B, Shmulevich I, Trayanova N. Digital twins in medicine. Nat Comput Sci. 2024;4(3):184–91. doi: 10.1038/s43588-024-00607-6 38532133 PMC11102043

[49] Clerx M, Collins P, de Lange E, Volders PGA. Myokit: A simple interface to cardiac cellular electrophysiology. Prog Biophys Mol Biol. 2016;120(1–3):100–14. doi: 10.1016/j.pbiomolbio.2015.12.008 26721671

[50] Hindmarsh AC, Brown PN, Grant KE, Lee SL, Serban R, Shumaker DE, et al. SUNDIALS: Suite of nonlinear and differential/algebraic equation solvers. ACM Trans Math Softw. 2005;31(3):363–96. doi: 10.1145/1089014.1089020

