# Supplementary material for: Phase resetting in human stem cell derived cardiomyocytes explains complex cardiac arrhythmias
Source: PLoS Comput Biol. 2026 Feb 4;22(2):e1013935. doi: 10.1371/journal.pcbi.1013935 (PMC12900431; doi:10.1371/journal.pcbi.1013935)
Supplement: S1 Text — (PDF) [file pcbi.1013935.s001.pdf]

S1 Text: Fitting a polynomial equation to experimental PRCs

We studied entrainment and phase-resetting in six spheroid pairs. Their PRCs are shown in Fig. S1. We model the PRCs using the function

$$g(\phi) = \begin{cases} 1 & 0 \le \phi < \phi_1, \\ 1 + A(\phi - \phi_1)^4 & \phi_1 \le \phi < \phi_r, \\ B(\phi - \phi_2)^2 + C & \phi_r \le \phi < \phi_3, \\ 1 + S(\phi - 1) & \phi_3 \le \phi < 1, \end{cases} \tag{1}$$

which is a piecewise-defined polynomial equation designed to be as simple as possible while capturing the main features observed in the experimental PRCs. The parameters A, ϕ_r, B and S are fit to the experimental data (see S1 Table) using a nonlinear least-squares optimization algorithm (Trust Region Reflective method (*1*)). Specifically, A controls the magnitude of the delay branch, ϕ_r defines the point of discontinuity between delay and advance, B controls the curvature of the hook in the initial portion of the advance branch, and S is the slope of the later portion of the advance branch. The parameters ϕ_1, ϕ_2, ϕ_3 and C are auxiliary: they are not fit directly but impose structural constraints and ensure continuity and smoothness. We set $\phi_1 = \phi_r - 0.25$ and $\phi_2 = \phi_r + 0.04$. The parameters ϕ_3 and C are uniquely determined to ensure continuity of both the function and its derivative at $\phi = \phi_3$, given by

$$\phi_3 = \phi_2 + S/(2B), \tag{2}$$

$$C = 1 - S(1 - \phi_3) - B(\phi_3 - \phi_2)^2. \tag{3}$$

The PRC shown in S1A Fig was used for the analysis in the main text. All six datasets showed qualitatively similar features: no resetting at early phases, a delay at intermediate phases, and apparent discontinuity leading to an advance at later phases. A hook in the early portion of the advancing branch was also visible in each dataset. The most prominent variation among the spheroids was the point of discontinuity ϕ_r. The hiPSC-CM spheroids in panels A–C have later points of discontinuity (0.71, 0.57, and 0.57, respectively) compared to those in panels D–F (0.42, 0.40, and 0.38, respectively). This suggests that the latter group is more readily entrained, possibly

due to stronger effective stimuli. The magnitude of the stimulus influences the PRC (*2*). It is likely that variability in the contact area between the ChR2-HEK and hiPSC-CM spheroids, as well as differences in spheroid size, contributed to these variations even under constant pulse duration.

References

1. T. F. Coleman, Y. Li, An interior trust region approach for nonlinear minimization subject to bounds. *SIAM Journal on optimization* **6** (2), 418–445 (1996).

2. M. R. Guevara, A. Shrier, L. Glass, Phase resetting of spontaneously beating embryonic ventricular heart cell aggregates. *American Journal of Physiology-Heart and Circulatory Physiology* **251** (6), H1298–H1305 (1986).

3. M. Courtemanche, L. Glass, M. D. Rosengarten, A. Goldberger, Beyond pure parasystole: promises and problems in modeling complex arrhythmias. *American Journal of Physiology-Heart and Circulatory Physiology* **257** (2), H693–H706 (1989).

4. F. Santoro, *et al.*, Ventricular fibrillation triggered by PVCs from papillary muscles: clinical features and ablation. *Journal of cardiovascular electrophysiology* **25** (11), 1158–1164 (2014).

5. T. Bury, *et al.*, The inverse problem for cardiac arrhythmias. *Chaos: An Interdisciplinary Journal of Nonlinear Science* **33** (12) (2023).

6. K. Takayanagi, *et al.*, Ectopic cycle length estimation from the quantified distribution patterns of ventricular bigeminy and trigeminy. *Heart Rhythm O2* **2** (2), 138–148 (2021).
