# Supplementary material for: Phase resetting in human stem cell derived cardiomyocytes explains complex cardiac arrhythmias
Source: PLoS Comput Biol. 2026 Feb 4;22(2):e1013935. doi: 10.1371/journal.pcbi.1013935 (PMC12900431; doi:10.1371/journal.pcbi.1013935)
Supplement: S2 Text — (PDF) [file pcbi.1013935.s002.pdf]

S2 Text: Parasystole and resetting in clinical ECGs

Selection criteria and preprocessing of Holter ECG recordings from patients

We have multi-day Holter recordings from 53 patients enrolled in the British Columbia PVC Registry, all of whom exhibit > 5% premature ventricular complexes (PVCs). Patients were included for further analysis according to the criteria shown in S2 Fig. For consistency with our modeling assumptions, we included only those patients whose PVCs were mostly of a single morphology, consistent with PVCs originating from a single ectopic focus. Additionally, we flagged patients as potential candidates for parasystole by applying a mask to identify coupling intervals that systematically "march in" or "march out."

Mask to identify cycling coupling intervals. To identify dynamics consistent with parasystole, we designed a rule-based mask to detect sequences in which the coupling interval progressively lengthens ("marching in") or shortens ("marching out"). A sequence was classified as *marching in* if it satisfied all of the following criteria:

1. alternating ectopic and sinus beats;
2. coupling intervals that progressively lengthen;
3. compensatory pauses that progressively shorten;
4. two consecutive sinus beats that terminate the sequence of alternating ectopic and sinus beats.

Similarly, a sequence was classified as *marching out* if it satisfied:

1. alternating ectopic and sinus beats;
2. coupling intervals that progressively shorten;
3. compensatory pauses that progressively lengthen;
4. two consecutive sinus beats prior to the sequence of alternating ectopic and sinus beats.

These masks were applied to each patient record to isolate segments exhibiting cycling coupling intervals suggestive of parasystole. Representative examples of both "marching in" and "marching out" patterns are shown in S3 Fig. A patient was selected for further analysis if their record contained at least three consecutive sequences of marching in or marching out. Based on this criterion, 7 patients (13% of the cohort) were included for further analysis.

Mathematical model for modulated parasystole

An early model for modulated parasystole. We build on an early model for modulated parasystole by Courtemanche et al. (*3*). This model assumes a sinus pacemaker with period t_s, an ectopic pacemaker with period t_e a refractory period θ, and a phase response curve for the ectopic pacemaker $g(\phi)$. The ectopic beat is blocked if it lands within the refractory period of the sinus beat. The sinus beat following an ectopic beat is always blocked. The model is formulated as follows. Let ϕ_i be the phase of the sinus beat within the ectopic cycle. Then one can determine the context in which it occurred, as outlined below:

Phase Range	Beat Sequence	Description
$0 \leq \phi < \frac{t_s-\theta}{t_e}$	V(N)	The sinus beat is blocked due to a preceding ectopic beat.
$\frac{t_s-\theta}{t_e} < \phi < \frac{t_s}{t_e}$	(V)N	The sinus beat is expressed and preceded by a blocked ectopic beat.
$\frac{t_s}{t_e} \leq \phi < 1$	N	The sinus beat is expressed.

If a sinus beat is blocked, there is no modulation of the ectopic pacemaker. The next phase is then obtained by adding the normalized sinus period t_s/t_e. If a sinus beat is expressed, there is modulation of the ectopic pacemaker, resulting in a shift of the phase by $1 - g(\phi)$. This yields the following difference equation for the phase

$$\phi_{i+1} = \begin{cases} \phi_i + \frac{t_s}{t_e} \pmod 1 & 0 \leq \phi_i < \frac{t_s-\theta}{t_e}, \\ \phi_i + \frac{t_s}{t_e} + 1 - g(\phi) \pmod 1 & \frac{t_s-\theta}{t_e} \leq \phi_i < 1, \end{cases} \tag{4}$$

where $0 \leq \phi_i < 1$.

Extension to include a conduction time into and out of the ectopic focus. Electrophysiological studies have reported a conduction delay between the initial firing of the ectopic focus and the subsequent PVC (*4*). We denote this delay as t_{out}. Similarly, we assume a conduction delay between a sinus beat and the resetting of the ectopic pacemaker, which we denote t_{in} (S4 Fig). Assumption of these conduction delays requires modification to the model equations. Let ϕ be the phase of the

sinus beat in the ectopic cycle as measured by the timing of the ectopic and sinus beat on an ECG (S4 Fig). Now, the elapsed time between firing of the focus and resetting of the focus is

$$t' = t_{\text{out}} + \phi t_e + t_{\text{in}} \tag{5}$$

which corresponds to a phase in the cycle of the ectopic pacemaker of

$$\phi' = \frac{t'}{t_e} = \phi + \frac{t_{\text{lag}}}{t_e} \tag{6}$$

where $t_{\text{lag}} = t_{\text{in}} + t_{\text{out}}$. To incorporate this property into the model, we modify the argument of the phase response curve accordingly. Other aspects remain unchanged. The model with conduction delay is given by

$$\phi_{i+1} = \begin{cases} \phi_i + \frac{t_s}{t_e} \pmod 1 & 0 \le \phi_i < \frac{t_s-\theta}{t_e}, \\ \phi_i + \frac{t_s}{t_e} + 1 - g\left(\phi + \frac{t_{\text{lag}}}{t_e}\right) \pmod 1 & \frac{t_s-\theta}{t_e} \le \phi_i < 1, \end{cases} \tag{7}$$

where $0 \le \phi_i < 1$.

Model variant that permits interpolated beats. Previous models (*3, 5*) assume that the sinus beat immediately following an ectopic beat is always blocked. This is not always the case in clinical data, where a patient can experience an interpolated PVC—a PVC that does not lead to a block of the subsequent sinus beat. In this model variant, we assume that the sinus beat is only blocked if it occurs during a refractory period following the ectopic beat. We take this refractory period to be θ, the same value that is assigned to the refractory period of the sinus beats.

Note that if $t_s < 2\theta$, a sinus beat following an ectopic necessarily lands in the refractory period. Therefore, for this range of sinus rates, the model is identical to Eqn. (7). However, for slower sinus rates $t_s > 2\theta$, there are four different outcomes depending on the phase, outlined below:

Phase Range	Beat Sequence	Description
$0 \le \phi < \frac{\theta}{t_e}$	V(N)	An ectopic beat followed by a blocked sinus beat.
$\frac{\theta}{t_e} \le \phi < \frac{t_s-\theta}{t_e}$	VN	An (interpolated) ectopic beat followed by a sinus beat.
$\frac{t_s-\theta}{t_e} \le \phi < \frac{t_s}{t_e}$	(V)N	A blocked ectopic beat followed by a sinus beat.
$\frac{t_s}{t_e} \le \phi < 1$	N	An additional sinus beat in the ectopic cycle

In this model, phase resetting only occurs for $\phi \geq \min(\theta/t_e, (t_s-\theta)/t_e)$. The difference equation model that permits interpolated beats is

$$\phi_{i+1} = \begin{cases} \phi_i + \frac{t_s}{t_e} \pmod{1} & 0 \leq \phi_i < \min\left(\frac{\theta}{t_e}, \frac{t_s-\theta}{t_e}\right), \\ \phi_i + \frac{t_s}{t_e} + 1 - g\left(\phi + \frac{t_{\text{lag}}}{t_e}\right) \pmod{1} & \min\left(\frac{\theta}{t_e}, \frac{t_s-\theta}{t_e}\right) \leq \phi_i < 1, \end{cases} \tag{8}$$

where $0 \leq \phi_i < 1$.

Construction of PRC from clinical ECG

We use segments of bigeminy (a single sinus beat between two ectopic beats) and trigeminy (two sinus beats between two ectopic beats) to construct an approximation of the PRC from clinical data (*6*). Notation for beat-to-beat intervals is shown in S5 Fig. We make the following assumptions:(i) the intervening sinus beats occur during a single cycle of the ectopic pacemaker; (ii) during trigeminy, the second sinus beat shortens the ectopic cycle. We let g denote the PRC that maps beat intervals according to

$$\text{VV}_1 = g(\text{VN}_1). \tag{9}$$

Construction from bigeminy data. During bigeminy, we collect data in the form of $(\text{VN}_1, \text{VV}_1)$, which enables us to approximate g over a limited range of VN_1 values. In clinical records, this relationship is typically a positive linear trend. Assuming a linear form,

$$\text{VV}_1 = S * \text{VN}_1 + C, \tag{10}$$

we estimate the slope S and intercept C using linear regression. In addition, we assume that when $\text{VN}_1 = \text{VV}_0 - t_{\text{lag}}$, the sinus stimulus lands at the end of the ectopic cycle (recall S4 Fig), resulting in no resetting ($\text{VV}_1 = \text{VV}_0$). Under this condition

$$\text{VV}_0 = g(\text{VV}_0 - t_{\text{lag}}) \implies C = (1 - S)\text{VV}_0 + S\, t_{\text{lag}}, \tag{11}$$

which provides a relationship between VV_0 and t_{lag} determined from data.

Construction from trigeminy data. During trigeminy, we collect data in the form of $(\text{VN}_1, \text{VN}_2, \text{VV}_2)$. We would like to infer VV_1 from these data in order to uncover a wider range of the PRC. The first

sinus beat modifies the ectopic cycle length by $\Delta T = \mathrm{VV}_1 - \mathrm{VV}_0$. Resetting from the second sinus beat then satisfies

$$\mathrm{VV}_2 = g(\mathrm{VN}_2 - \Delta T) + \Delta T. \tag{12}$$

Assuming the second sinus beat shortens the ectopic cycle length according to Eqn. 10, we have

$$\mathrm{VV}_2 = S(\mathrm{VN}_2 - \Delta T) + C + \Delta T. \tag{13}$$

Substituting the expression for C from Eqn. 11 and rearranging yields

$$\mathrm{VV}_1 = \frac{\mathrm{VV}_2 - S(\mathrm{VN}_2 + t_{\mathrm{lag}})}{1 - S}. \tag{14}$$

Since we have estimates for S and t_{lag} (see below), and VV_2 and VN_2 are measured directly from trigeminy sequences, we can infer VV_1 and augment the dataset used to construct the PRC.

Phase response curve of the ectopic pacemaker. Above, we have written the phase response curve in terms of beat-to-beat intervals on the ECG. The phase response curve of the ectopic pacemaker can be obtained by rescaling to get the phase as follows:

$$\phi = \frac{\mathrm{VN}_1 + t_{\mathrm{lag}}}{t_e}. \tag{15}$$

Parameter estimation for the modulated parasystole model

The parameters of the modulated parasystole model are listed in S2 Table along with the methods used to estimate them from the clinical and experimental datasets. The sinus cycle length (t_s) is estimated as the mean interbeat interval in a 30-second section of a recording. The ectopic cycle length (t_e), the lag time (t_{lag}) and PRC discontinuity (ϕ_r) are determined by fitting the model to a 30-second segment of data identified as exhibiting a cycling coupling interval (S3 Fig). These parameters are chosen to minimize the mean absolute error between the clinical and model-generated interbeat intervals. The PRC resetting slope (S) is determined by the slope in the plot of VV intervals against VN intervals (S6 Fig). The refractory period (θ) is determined from the lower bound of NV intervals at each heart rate (S7 Fig). The remaining parameters (m, p, r, N), which govern the shape of the PRC, are obtained from experimental data. Individual parameter values for each patient are reported in S3 Table .

Using the fitted parameter values, we evaluate the model's ability to reproduce other 30-second segments from the same recording. The analysis is restricted to segments that do not contain unidentified beats. In this evaluation, the model is not simulated with a fixed sinus cycle length (t_s), but instead with a sequence of sinus cycle lengths obtained from averaging the patient interbeat intervals over a rolling window of two beats. To assess goodness of fit, we use the two-tailed Kolmogorov-Smirnov test, which evaluates whether two distributions are significantly different. The model is considered a good fit if the Kolmogorov–Smirnov test does not detect a statistically significant difference between the interbeat interval distributions of the patient and the model ($p > 0.05$). The proportion of 30-second segments that meet this criterion is reported in S3 Table.

References

1. T. F. Coleman, Y. Li, An interior trust region approach for nonlinear minimization subject to bounds. *SIAM Journal on optimization* **6** (2), 418–445 (1996).

2. M. R. Guevara, A. Shrier, L. Glass, Phase resetting of spontaneously beating embryonic ventricular heart cell aggregates. *American Journal of Physiology-Heart and Circulatory Physiology* **251** (6), H1298–H1305 (1986).

3. M. Courtemanche, L. Glass, M. D. Rosengarten, A. Goldberger, Beyond pure parasystole: promises and problems in modeling complex arrhythmias. *American Journal of Physiology-Heart and Circulatory Physiology* **257** (2), H693–H706 (1989).

4. F. Santoro, *et al.*, Ventricular fibrillation triggered by PVCs from papillary muscles: clinical features and ablation. *Journal of cardiovascular electrophysiology* **25** (11), 1158–1164 (2014).

5. T. Bury, *et al.*, The inverse problem for cardiac arrhythmias. *Chaos: An Interdisciplinary Journal of Nonlinear Science* **33** (12) (2023).

6. K. Takayanagi, *et al.*, Ectopic cycle length estimation from the quantified distribution patterns of ventricular bigeminy and trigeminy. *Heart Rhythm O2* **2** (2), 138–148 (2021).
