# Supplementary material for: Phase resetting in human stem cell derived cardiomyocytes explains complex cardiac arrhythmias
Source: PLoS Comput Biol. 2026 Feb 4;22(2):e1013935. doi: 10.1371/journal.pcbi.1013935 (PMC12900431; doi:10.1371/journal.pcbi.1013935)
Supplement: S1 Fig — The red line shows the fitted PRC function given by Eq. (1). Optimal parameters were obtained using a nonlinear least-squares algorithm (Trust Region Reflective method). Best-fit parameter values for each aggregate are listed in S1 Table. The inset shows the root mean squared error (RMSE) of each fit. (PDF) [file pcbi.1013935.s003.pdf]

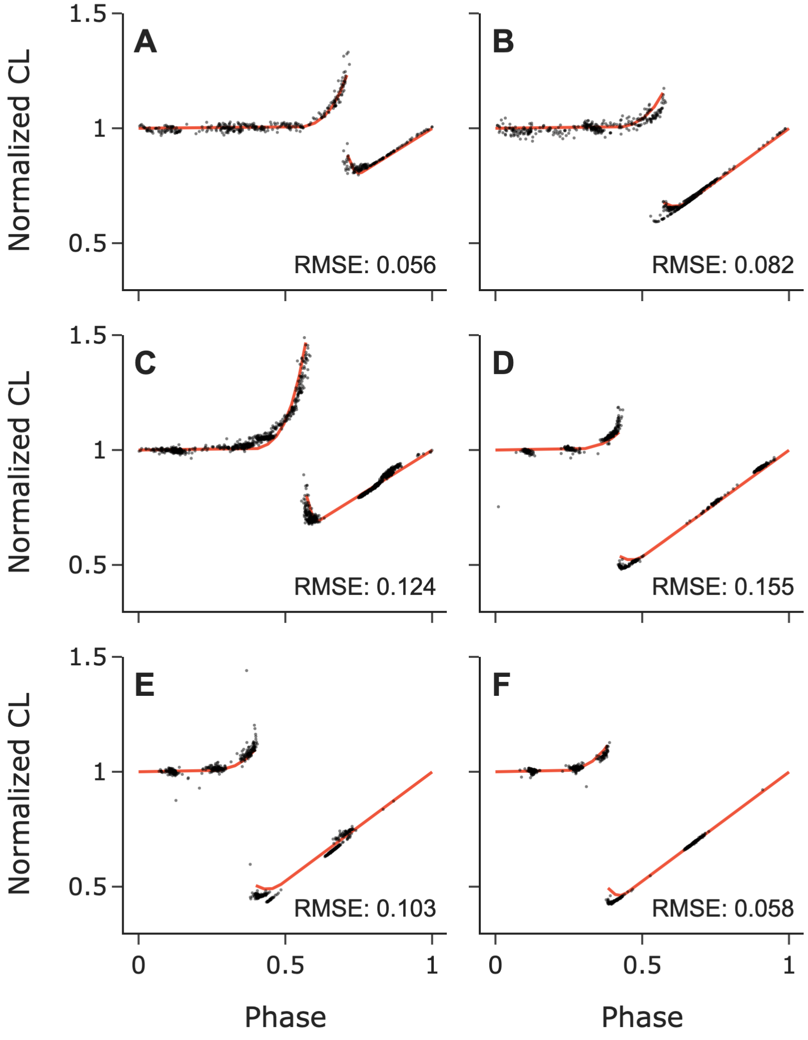

S1 Figure : **Phase-resetting curves for individual hiPSC-CM spheroids.** The red line shows the fitted PRC function given by Eqn. (1). Optimal parameters were obtained using a nonlinear least-squares algorithm (Trust Region Reflective method). Best-fit parameter values for each aggregate are listed in S1 Table. The inset shows the root mean squared error (RMSE) of each fit.
