# Supplementary material for: Phase resetting in human stem cell derived cardiomyocytes explains complex cardiac arrhythmias
Source: PLoS Comput Biol. 2026 Feb 4;22(2):e1013935. doi: 10.1371/journal.pcbi.1013935 (PMC12900431; doi:10.1371/journal.pcbi.1013935)
Supplement: S2 Fig — (PDF) [file pcbi.1013935.s004.pdf]

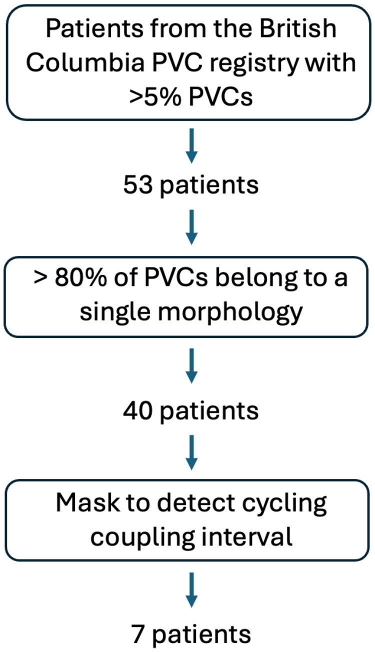

S2 Figure : Filtering of clinical data based on the presence of a dominant PVC morphology and a cycling coupling interval.
