# Supplementary material for: Phase resetting in human stem cell derived cardiomyocytes explains complex cardiac arrhythmias
Source: PLoS Comput Biol. 2026 Feb 4;22(2):e1013935. doi: 10.1371/journal.pcbi.1013935 (PMC12900431; doi:10.1371/journal.pcbi.1013935)
Supplement: S3 Fig — Each panel shows inter-beat intervals from six different patient records identified as exhibiting a cycling coupling interval. Intervals are color-coded as follows: blue for sinus–sinus, red for sinus–ectopic, green for ectopic–sinus, and purple for ectopic–ectopic beats. These sections are used to fit the model parameters te, tlag, and ϕr for each record by minimizing the mean absolute error between the model simulation and the data. (PDF) [file pcbi.1013935.s005.pdf]

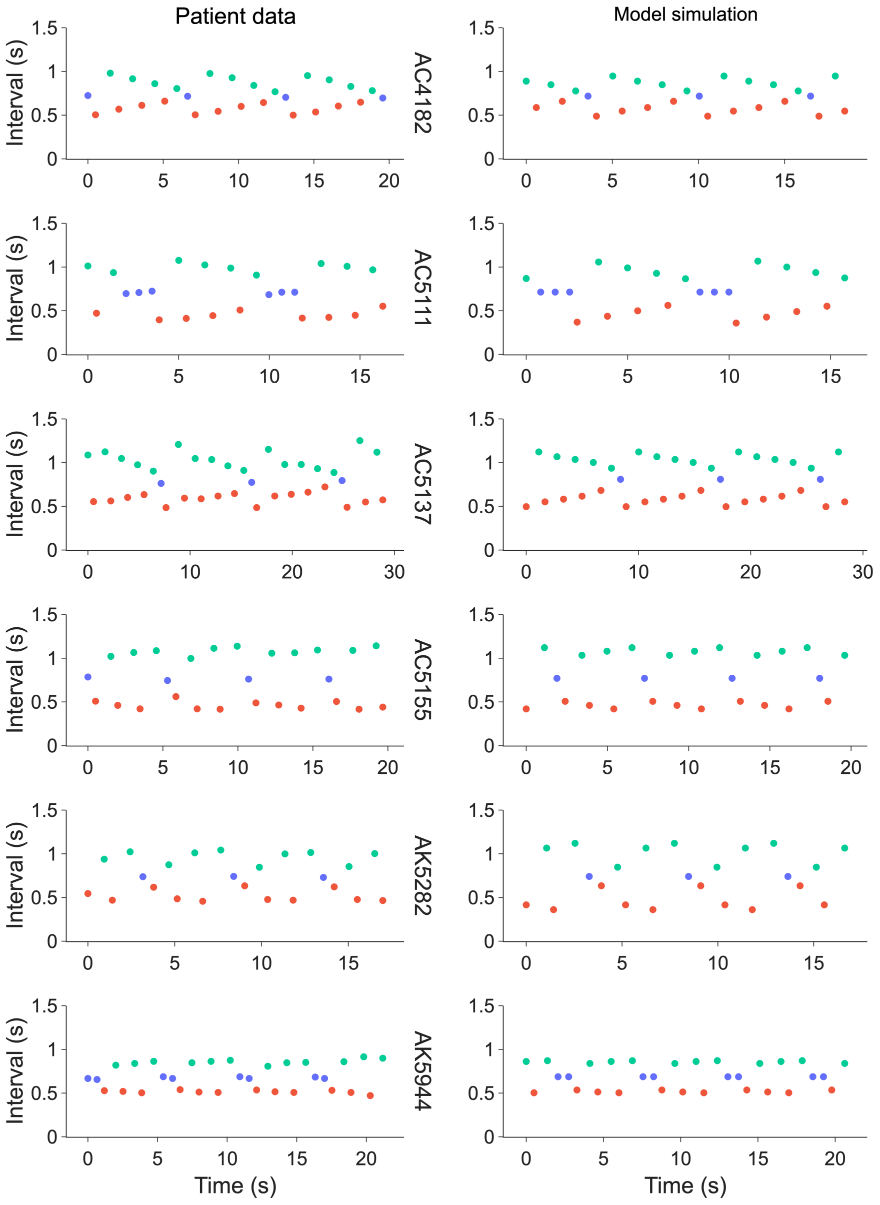

S3 Figure : **Sections of patient data with cycling coupling intervals (left) and corresponding model simulation (right).** Each panel shows inter-beat intervals from six different patient records identified as exhibiting a cycling coupling interval. Intervals are color-coded as follows: blue for sinus–sinus, red for sinus–ectopic, green for ectopic–sinus, and purple for ectopic–ectopic beats. These sections are used to fit the model parameters t_e, t_{lag}, and ϕ_r for each record by minimizing the mean absolute error between the model simulation and the data.
