# Supplementary material for: Phase resetting in human stem cell derived cardiomyocytes explains complex cardiac arrhythmias
Source: PLoS Comput Biol. 2026 Feb 4;22(2):e1013935. doi: 10.1371/journal.pcbi.1013935 (PMC12900431; doi:10.1371/journal.pcbi.1013935)
Supplement: S4 Fig — This ECG shows a bigeminal rhythm (alternating sinus beats and ectopic beats). Assuming the ectopic beats originate from an ectopic pacemaker, we define the phase ϕ as the time between the ectopic beat and the subsequent sinus beat divided by the natural period of the ectopic pacemaker te. We assume that there is a conduction time tout from the firing of the ectopic pacemaker to the time of the PVC.We also assume there is a conduction time t in from the time of the sinus beat to the modulation of the ectopic focus. The shifted phase ϕ + tlag/te therefore represents the phase at which sinus stimulus arrives at the ectopic focus. (PDF) [file pcbi.1013935.s006.pdf]

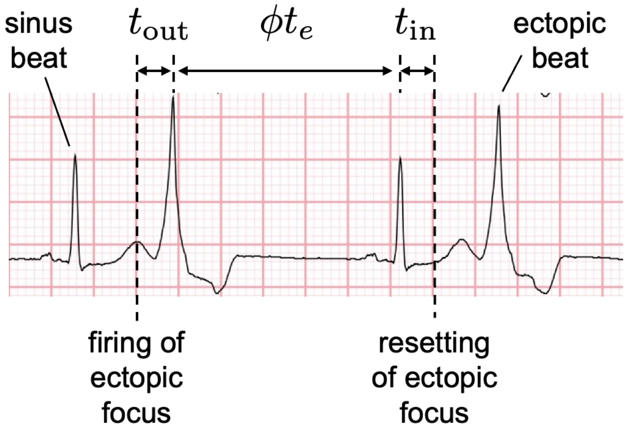

S4 Figure : **Illustration of conduction time into and out of the ectopic focus.** This ECG shows a bigeminal rhythm (alternating sinus beats and ectopic beats). Assuming the ectopic beats originate from an ectopic pacemaker, we define the phase ϕ as the time between the ectopic beat and the subsequent sinus beat divided by the natural period of the ectopic pacemaker t_e. We assume that there is a conduction time $t_{\rm out}$ from the firing of the ectopic pacemaker to the time of the PVC. We also assume there is a conduction time $t_{\rm in}$ from the time of the sinus beat to the modulation of the ectopic focus. The shifted phase $\phi + t_{\rm lag}/t_{\rm e}$ therefore represents the phase at which sinus stimulus arrives at the ectopic focus.
