# Supplementary material for: Phase resetting in human stem cell derived cardiomyocytes explains complex cardiac arrhythmias
Source: PLoS Comput Biol. 2026 Feb 4;22(2):e1013935. doi: 10.1371/journal.pcbi.1013935 (PMC12900431; doi:10.1371/journal.pcbi.1013935)
Supplement: S5 Fig — (A) Trace shows a section of ECG from record AC5137 during bigeminy (one sinus beat in between ectopic beats). VN1 denotes the interval from the ectopic beat to the (first) sinus beat. VV0 denotes the intrinsic ectopic cycle length. VV1 denotes modified ectopic cycle length due to the sinus beat. (B) Trace shows a section of the same record in trigeminy (two sinus beats in between ectopic beats). VN1 denotes the interval from the ectopic beat to the first sinus beat. VN2 denotes the interval from the ectopic beat to the second sinus beat. VV0 denotes the intrinsic ectopic cycle length. VV1 denotes modified ectopic cycle length due to the first sinus beat. VV2 denotes the modified cycle length due to the first and second sinus beats combined. (PDF) [file pcbi.1013935.s007.pdf]

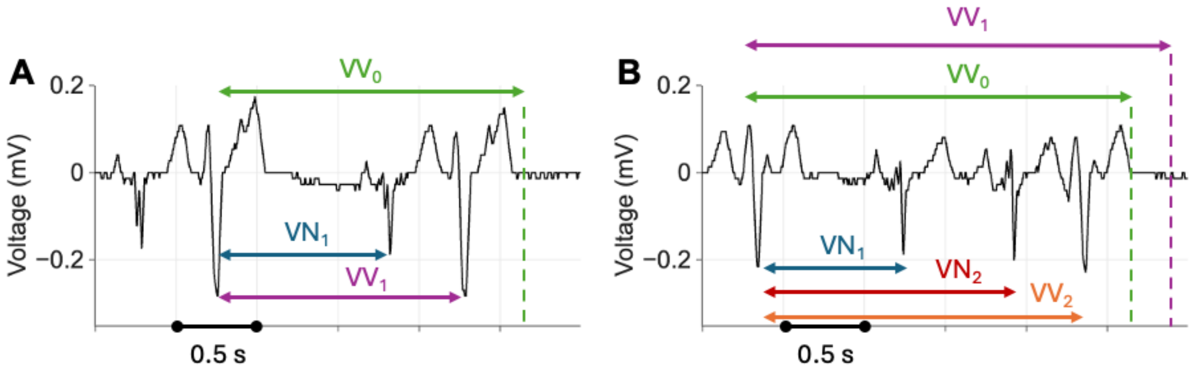

S5 Figure : Notation for beat-to-beat intervals. (A) Trace shows a section of ECG from record AC5137 during bigeminy (one sinus beat in between ectopic beats). VN_1 denotes the interval from the ectopic beat to the (first) sinus beat. VV_0 denotes the intrinsic ectopic cycle length. VV_1 denotes modified ectopic cycle length due to the sinus beat. (B) Trace shows a section of the same record in trigeminy (two sinus beats in between ectopic beats). VN_1 denotes the interval from the ectopic beat to the first sinus beat. VN_2 denotes the interval from the ectopic beat to the second sinus beat. VV_0 denotes the intrinsic ectopic cycle length. VV_1 denotes modified ectopic cycle length due to the first sinus beat. VV_2 denotes the modified cycle length due to the first and second sinus beats combined.
