# Supplementary material for: Phase resetting in human stem cell derived cardiomyocytes explains complex cardiac arrhythmias
Source: PLoS Comput Biol. 2026 Feb 4;22(2):e1013935. doi: 10.1371/journal.pcbi.1013935 (PMC12900431; doi:10.1371/journal.pcbi.1013935)
Supplement: S6 Fig — (A) Inter-beat intervals for a 30 s section of ECG from record AC5137. Intervals are either between two sinus beats (blue), between a sinus and an ectopic beat (red) or between an ectopic and sinus beat (green). (B) The interval between two consecutive ectopic beats (VV) as a function of the interval between the first ectopic and intervening sinus beat (VN) during periods of NIB = 1 (a single sinus beat between two ectopic beats). The slope of the linear regression through the points provides an estimation for the slope of the resetting curve (S). (PDF) [file pcbi.1013935.s008.pdf]

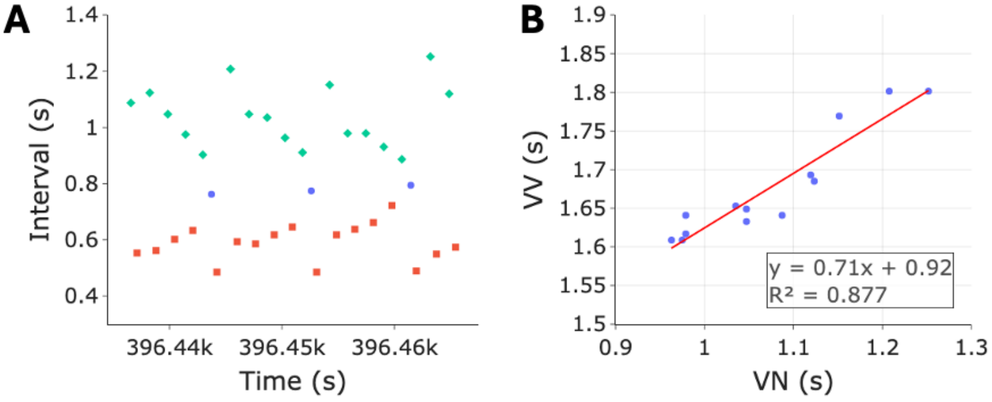

S6 Figure : **Estimating the PRC resetting slope (S) from the ECG.** (A) Inter-beat intervals for a 30 s section of ECG from record AC5137. Intervals are either between two sinus beats (blue), between a sinus and an ectopic beat (red) or between an ectopic and sinus beat (green). (B) The interval between two consecutive ectopic beats (VV) as a function of the interval between the first ectopic and intervening sinus beat (VN) during periods of NIB=1 (a single sinus beat between two ectopic beats). The slope of the linear regression through the points provides an estimation for the slope of the resetting curve (S).
