# Supplementary material for: Phase resetting in human stem cell derived cardiomyocytes explains complex cardiac arrhythmias
Source: PLoS Comput Biol. 2026 Feb 4;22(2):e1013935. doi: 10.1371/journal.pcbi.1013935 (PMC12900431; doi:10.1371/journal.pcbi.1013935)
Supplement: S7 Fig — (A) Distributions of the coupling interval (NV) at different values of the sinus cycle length (bins of width 0.1 s) for record AC5137. (B) Linear regression through the points that mark the 5th percentile of each distribution. The refractory period at a given sinus cycle length is taken as the coupling interval on this linear regression minus a fixed amount ϵ = 0.05 s. Let m and c be the slope and intercept of the linear regression, then (ts) = (ts − c)/m − ϵ. (PDF) [file pcbi.1013935.s009.pdf]

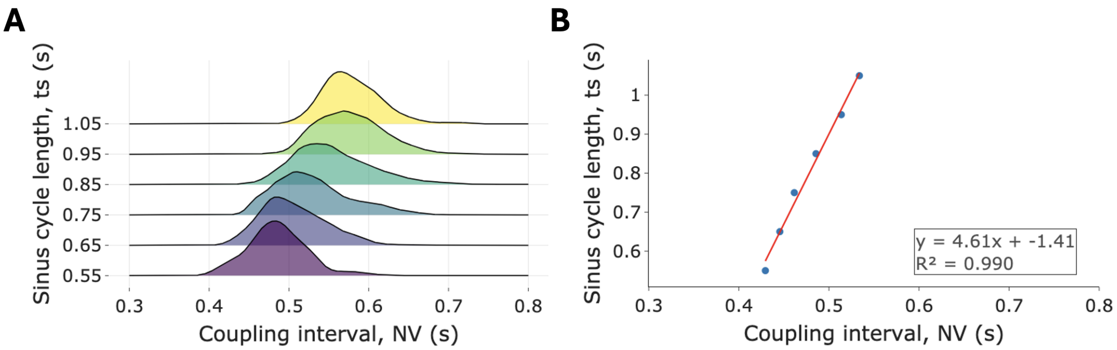

S7 Figure : **Estimating the refractory period (θ) from the ECG**. (A) Distributions of the coupling interval (NV) at different values of the sinus cycle length (bins of width 0.1 s) for record AC5137. (B) Linear regression through the points that mark the 5th percentile of each distribution. The refractory period at a given sinus cycle length is taken as the coupling interval on this linear regression minus a fixed amount $\epsilon = 0.05$ s. Let m and c be the slope and intercept of the linear regression, then $\theta(t_s) = (t_s - c)/m - \epsilon$.
