# Supplementary material for: Phase resetting in human stem cell derived cardiomyocytes explains complex cardiac arrhythmias
Source: PLoS Comput Biol. 2026 Feb 4;22(2):e1013935. doi: 10.1371/journal.pcbi.1013935 (PMC12900431; doi:10.1371/journal.pcbi.1013935)
Supplement: S8 Fig — (A) Poincare´ map from Fig 3B for 𝜏 = 0.66, showing a stable cycle of period 3. At this phase-locking pattern, the stimulus phase in the oscillator’s cycle alternates between ϕ1, ϕ2, and ϕ3. (B) Signal trace for the same experiment, showing the three phases of the stimulus in the phase-locked pattern. (C) Corresponding interbeat intervals, with the same notation as in Figs 1D and 2C. (PDF) [file pcbi.1013935.s010.pdf]

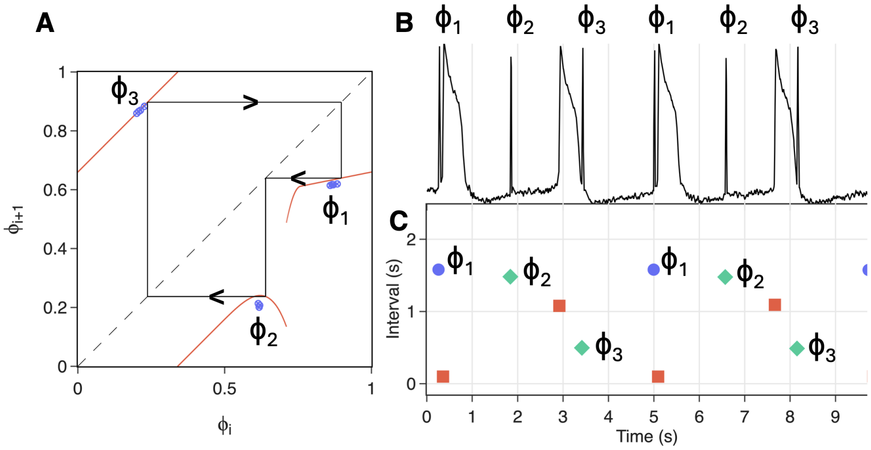

S8 Figure : **Notation in Poincaré Maps.** (**A**) Poincaré map from Fig. 3B for $\tau = 0.66$, showing a stable cycle of period 3. At this phase-locking pattern, the stimulus phase in the oscillator's cycle alternates between ϕ_1, ϕ_2, and ϕ_3. (**B**) Signal trace for the same experiment, showing the three phases of the stimulus in the phase-locked pattern. (**C**) Corresponding interbeat intervals, with the same notation as in Figs. 1D and 2C.
