# Supplementary material for: Phase resetting in human stem cell derived cardiomyocytes explains complex cardiac arrhythmias
Source: PLoS Comput Biol. 2026 Feb 4;22(2):e1013935. doi: 10.1371/journal.pcbi.1013935 (PMC12900431; doi:10.1371/journal.pcbi.1013935)
Supplement: S9 Fig — (B) stimulus strength from 0.05 nA in indigo to 0.19 nA in green, stimulus duration = 10 ms, temperature = 37°C. (C) stimulus strength = 0.15 nA, stimulus duration from 5 ms in indigo to 20 ms in blue, temperature = 37°C. (PDF) [file pcbi.1013935.s011.pdf]

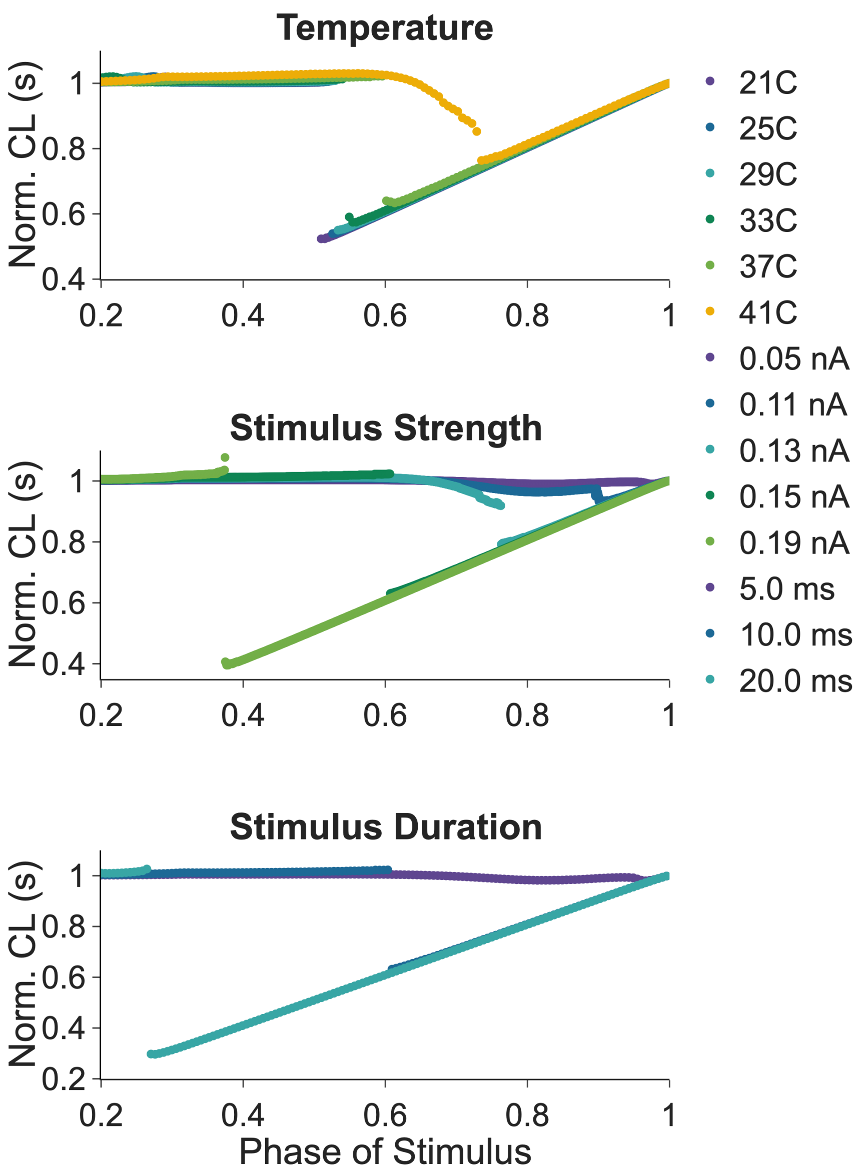

S9 Figure : **Effects of temperature, stimulus strength, and duration on the PRC from the Paci et al. 2020 ionic model** (**A**) Stimulus strength = 0.15 nA, stimulus duration = 10 ms, temperature from 21°C in indigo to 41°C in yellow. (**B**) stimulus strength from 0.05 nA in indigo to 0.19 nA in green, stimulus duration = 10 ms, temperature = 37°C. (**C**) stimulus strength = 0.15 nA, stimulus duration from 5 ms in indigo to 20 ms in blue, temperature = 37°C.
