# Supplementary material for: Phase resetting in human stem cell derived cardiomyocytes explains complex cardiac arrhythmias
Source: PLoS Comput Biol. 2026 Feb 4;22(2):e1013935. doi: 10.1371/journal.pcbi.1013935 (PMC12900431; doi:10.1371/journal.pcbi.1013935)
Supplement: S1 Table — The intrinsic cycle length (iCL) represents the range of spontaneous beat intervals. (PDF) [file pcbi.1013935.s012.pdf]

Aggregate	A	ϕ_r	B	S	RMSE	iCL (s)
A	60	0.71	50	0.80	0.056	2.07 - 2.41
B	40	0.57	10	0.93	0.082	1.99 - 3.07
C	120	0.57	0.73	0.80	0.124	0.61 - 0.80
D	20	0.42	10	0.93	0.155	1.13 - 1.29
E	25	0.40	10	0.95	0.103	1.32-1.37
F	30	0.38	20	0.95	0.058	1.36 - 1.41

S1 Table: Optimal parameters for fitting the PRC function (Eqn. (1)) to the experimental PRCs shown in Fig. S1. Aggregate labels correspond to the panel labels in Fig. S1. The root mean squared error (RMSE) is used to assess the goodness of fit. The intrinsic cycle length (iCL) represents the range of spontaneous beat intervals.
