# Supplementary material for: Phase resetting in human stem cell derived cardiomyocytes explains complex cardiac arrhythmias
Source: PLoS Comput Biol. 2026 Feb 4;22(2):e1013935. doi: 10.1371/journal.pcbi.1013935 (PMC12900431; doi:10.1371/journal.pcbi.1013935)
Supplement: S2 Table — Ranges/values are across the 7 patients identified as having cycling coupling intervals given in S3 Table. PRC: phase response curve; s: seconds; –: dimensionless. (PDF) [file pcbi.1013935.s013.pdf]

Label	Definition	Units	Range/Value	Estimation method
t_s	sinus cycle length	s	0.36-1.61	Average of RR intervals within a 30 s window
t_e	ectopic cycle length	s	1.50-2.20	Fit to ECG data (Fig. S3)
t_{lag}	time between sinus beat and resetting of ectopic focus	s	0.35-0.48	Fit to ECG data (Fig. S3)
θ	refractory period	s	0.25-0.68	Lower bound of NV intervals at each heart rate (Fig. S7)
S	PRC resetting slope	–	0.05-0.95	Slope of linear regression of VV-VN plot (Fig. S6)
ϕ_r	PRC discontinuity	–	0.40-0.80	Fit to ECG data (Fig. S3)
m	PRC delay quotient	–	45	Obtained from experimental data
p	PRC delay exponent	–	2.5	Obtained from experimental data
r	PRC delay asymptote	–	0.745	Obtained from experimental data
N	PRC hill coefficient	–	35	Obtained from experimental data

S2 Table: Parameters, definitions, and estimation methods for the modulated parasystole model. Ranges/values are across the 7 patients identified as having cycling coupling intervals given in Table S3. PRC: phase response curve; s: seconds; –: dimensionless.
