# Supplementary material for: Phase resetting in human stem cell derived cardiomyocytes explains complex cardiac arrhythmias
Source: PLoS Comput Biol. 2026 Feb 4;22(2):e1013935. doi: 10.1371/journal.pcbi.1013935 (PMC12900431; doi:10.1371/journal.pcbi.1013935)
Supplement: S3 Table — The sinus cycle length (ts) is measured for each 30-second section of the record and given as a range. The refractory period (θ) is determined from a linear function of ts, derived from the distribution of coupling intervals across heart rates (S7 Fig). The ectopic cycle length (te), the lag time (tlag), and the PRC discontinuity (ϕ𝑟) are determined by fitting the model to a 30-second section identified as having cycling coupling intervals. The slope of the PRC (S) is determined by the slope in the plot of VV intervals against VN intervals (S6 Fig). The model is considered a good fit if the Kolmogorov–Smirnov test does not detect a statistically significant difference (p > 0.05) between the interbeat interval distributions of the patient and the model. (PDF) [file pcbi.1013935.s014.pdf]

Record ID	t_s (s)	θ (s)	t_e (s)	t_{lag} (s)	S	ϕ_r	Prop. record fit (± 5%/10%)
AC4182	0.46-1.10	0.40-0.48	1.88	0.44	0.80	0.66	75.5% / 99.7%
AC5111	0.36-1.29	0.25-0.45	1.51	0.25	0.08	0.75	63.4% / 100.0%
AC5137	0.50-1.28	0.35-0.47	2.00	0.46	0.80	0.71	72.2% / 100.0%
AC5155	0.42-1.30	0.33-0.41	1.50	0.46	0.93	0.48	73.2% / 95.3%
AK5282	0.52-1.14	0.40-0.50	1.61	0.30	0.75	0.41	95.0% / 99.8%
AK5942	0.73-1.61	0.64-0.68	2.20	0.48	0.60	0.40	81.3% / 92.3%
AK5944	0.53-1.27	0.42-0.52	1.50	0.41	0.59	0.40	63.4% / 89.3%

S3 Table: Model parameter values determined for each patient, along with the proportion of 30-second segments of the ECG record that are well described by the model within a ±5% and ±10% variation in parameter values. The sinus cycle length (t_s) is measured for each 30-second section of the record and given as a range. The refractory period (θ) is determined from a linear function of t_s, derived from the distribution of coupling intervals across heart rates (Fig. S7). The ectopic cycle length (t_e), the lag time (t_{lag}), and the PRC discontinuity (ϕ_r) are determined by fitting the model to a 30-second section identified as having cycling coupling intervals. The slope of the PRC (S) is determined by the slope in the plot of VV intervals against VN intervals (Fig. S6). The model is considered a good fit if the Kolmogorov–Smirnov test does not detect a statistically significant difference ($p > 0.05$) between the interbeat interval distributions of the patient and the model.
